# Judgement bias of group housed gestating sows predicted by behavioral traits, but not physical measures of welfare

**DOI:** 10.1371/journal.pone.0264258

**Published:** 2022-02-25

**Authors:** Kristina M. Horback, Thomas D. Parsons

**Affiliations:** 1 Department of Animal Science, University of California, Davis, CA, United States of America; 2 Department of Clinical Studies, Swine Teaching and Research Center, University of Pennsylvania School of Veterinary Medicine, Kennett Square, PA, United States of America; Institut de Recerca i Estudis en Primatologia, SPAIN

## Abstract

Judgement bias testing has emerged as a potential tool for assessing affective states in animals. Researchers infer an animal’s affective state based on an animal’s response to an ambiguous stimulus that is intermediate to both the rewarded and punished conditioned stimuli. Animals can be classified as “optimistic” or having a positive affective state if the animal displays behaviors that suggest an increased expectation of reward in the face of ambiguous stimuli. Alternatively, animals can be classified “pessimistic” or having a negative affective state if the animal displays behaviors that suggest an increased expectation of punishment in the face of ambiguous stimuli. Recent reports in multiple species question what factors influence performance in judgement bias testing, and which may allow for erroneous conclusions regarding individual affective state. In order to better understand this concern, 25 female swine were subjected to behavioral assessments at critical rearing stages to determine response variability. These same individuals were then assessed for physical measures of welfare and judgement bias using the “go/no-go” task as breeding adults. Sows which were more aggressive approached the ambiguous, but not the positive, stimulus significantly faster than others. Both optimistic and pessimistic biases were observed despite all sows living in enriched housing, and, sows with more positive physical welfare measures (fewer skin lesions and healthy body condition) did not exhibit more optimistic judgement biases. Our data demonstrate that behavior traits, such as *aggressiveness*, can affect a sow’s performance in a judgement bias test, while measures of physical health did not. We suggest that individual differences in behavior (e.g., bold-aggressive behavioral syndrome, or, proactive coping style) generate different emotional responses and can contribute to the animal’s overall affective state more so than physical ailment. Our findings highlight the complexity of how different factors impact an animal’s overall affective state and support the need for complementary measures in future JBT studies, including personality assessment.

## Introduction

Due to both government legislation and consumer demands, limitations have been established on the use of individual stalls to house gestating gilts and sows in many developed countries, including the EU [1], Australia, New Zealand, Canada, South Africa and ten states in the United States [2,3]. Group housing sows in pens for the majority of their gestation period allows for a greater freedom of movement, more opportunities to express species-specific behavior, and a more complex social environment. On the other hand, it can also lead to increases in injuries and lameness caused by social aggression [4,5]. Researchers often assess the welfare of individual sows in a pen by using physical metrics of poor welfare (i.e., skin lesions, lameness, or body condition), with little attention being given to the subjective psychological experience of the sows (i.e., pain/pleasure, affective state). It is particularly imperative to examine this latter issue in swine breeding animals (e.g., parent stock) as they live much longer lives than market hogs and could potentially suffer long-term negative emotional states.

The evaluation of the emotional states in non-human animals rely on physiological and behavioral indicators of arousal, as well as measures of conditioned responses to infer pain or pleasure experience (e.g., motivation and aversion testing) [6]. This evaluation can be expanded to include the cognitive component of an emotional state, such as, the impact of emotions on information processing (e.g., attention, memory, and judgement/appraisal) [7,8]. Research on human and non-human animals indicates that negative mental states, such as anxiety or depression, can induce pessimistic appraisals of ambiguous stimuli (i.e., expectation of punishment, or, lack of reward) [9,10]. For example, rats, piglets, and starlings in barren, crowded, or unpredictable housing conditions will display pessimistic cognitive bias in their appraisal of ambiguous stimuli, and thus, are inferred to experience more negative affective states [11–13]. While this assessment of ambiguous cue interpretation allows researchers to infer how an individual’s emotional state may influence information processing, it typically does not consider the influence of individual differences in behavioral response (i.e., personality) or individual sensitivity to reinforcer [14].

Personality can be described as characteristics of behavioral reactions such as intensity, speed, response threshold, latency and recovery time, which are most clearly expressed in novel and unpredictable situations, and are *relatively* stable throughout an individual’s development [15]. Given that personality is most clearly expressed in novel and unpredictable situations, behavioral tests are constructed to elicit high-arousal responses. Individual differences in personality may influence immunity strength [16], the performance of abnormal or stereotypic behaviors [17,18], and the expression of pain [19]. New research suggests that an animal’s personality can also influence the way in which stimuli are processed and evaluated; in other words, personality can shape judgement biases [20–27]. For example, proactive (exploratory and bold) grower pigs are more likely to display optimistic judgement biases than reactive (non-exploratory and cautious) grower pigs [20], dogs which receive higher traits scores for sociability, excitability and non-social-fear display more optimistic judgement biases than more fearful, less social dogs [21], and, neurotic orange-winged amazon parrots show a greater attention bias for environmental stimuli [22].

Given that breeding sows are the longest living population of swine in a production setting, it is important to understand the causes, mechanisms, and indicators of long-term negative emotional states to prevent possible suffering. As reviewed in a comprehensive book on the welfare of production swine [28], there is considerable attention in the literature given to the health and welfare of gestating and lactating sows as inferred from biological measurements and behavioral indicators. In contrast, to our knowledge, there has been only one study which utilizes cognitive methods (i.e., judgement bias paradigm) to infer affective state in gestating sows [29]. There are far more studies evaluating mood among younger animals, such as the investigation of stocking density impacting performance in judgment bias test among 14-week-old pigs [30], or the use of associative learning among 12-week-old pigs to evaluate emotional contagion [31]. In addition, while there have been approximately 48 studies on coping styles or personality traits among swine [32], less than a dozen specifically address consistent individual differences among breeding sows [33–38]. Given that there is limited information on the use and validity of judgement bias testing to evaluate affective states in sexually-mature sows, there is a need to investigate possible influences on performance in this cognitive task.

The aim of the present study is to investigate whether consistent individual differences in behavior of breeding sows predicts performance in a spatial discrimination judgement bias task. Based on recent reports linking animal personality, or coping style, to performance in a cognitive bias test, we predict that one or more behavioral traits evaluated will predict performance (e.g., response latency) in a spatial discrimination task. In addition, if judgement bias reflects the affective state of an animal (i.e., biological health and mental state), then it is important to test how physical measurements of welfare (i.e., skin lesions, body condition) also could impact performance in judgement bias task and contribute to mental well-being.

## Methods

### Animals and housing

**Fig 1:**
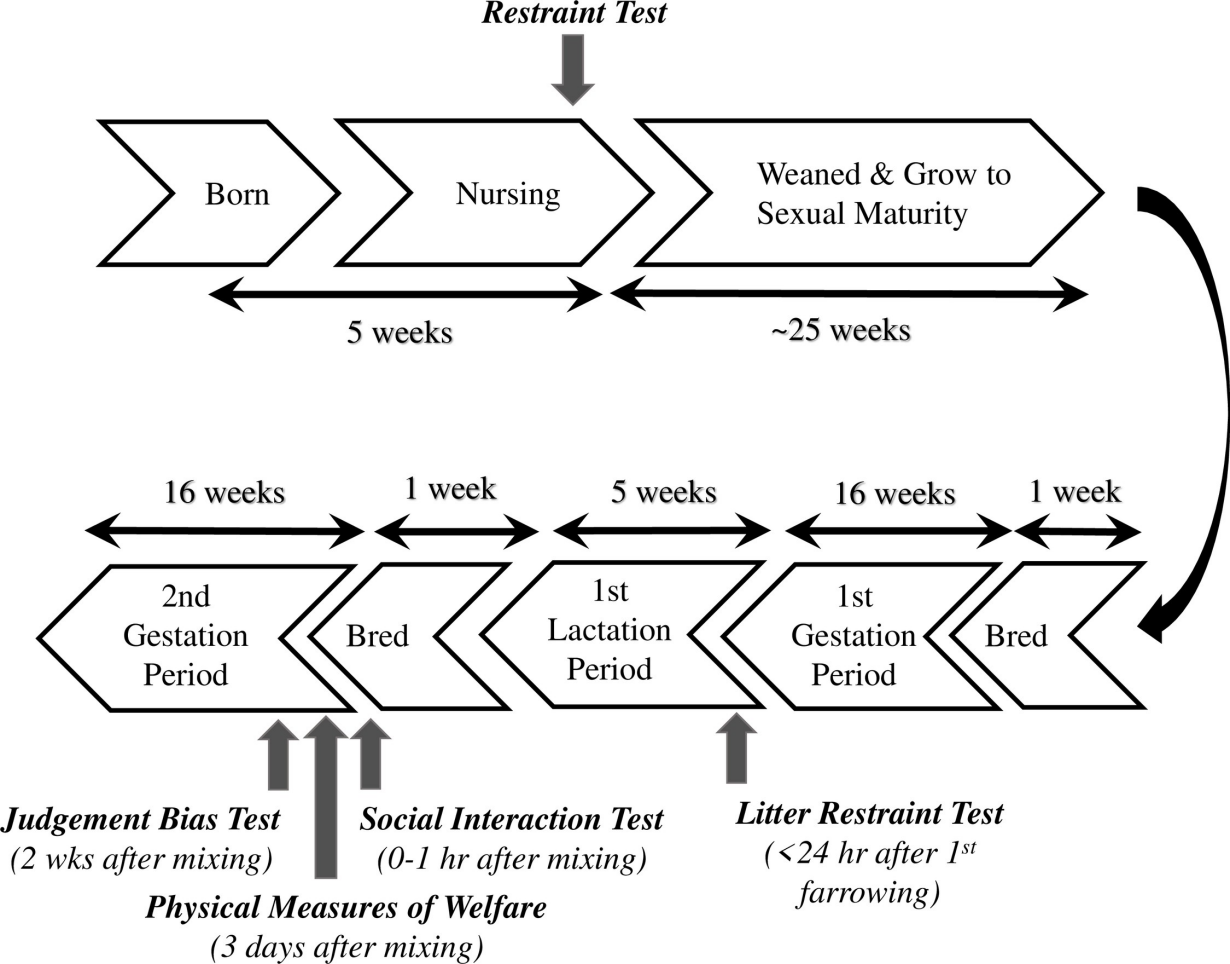
Timeline of data collection. Data collected at 5 weeks old (Restraint Test), 24 hours post-farrow (Litter Restraint Test), and, 1 hour (Social Interaction Test) and 2 weeks (Judgement Bias Test) post-mixing into group pen, as well how data collection corresponds to the life cycle of the sow.

Animals observed in this study were born and reared at the Swine Teaching and Research Center located at the School of Veterinary Medicine, University of Pennsylvania. At this research facility, approximately 120 to 130 gestating sows are housed in a dynamic, multiparous group pen and fed via electronic sow feeding stations (ESF, Schauer Agrotronic Compident 7). The sow pen allows for 2.4 m^2^ per sow, and includes nine 3.0 x 2.1 m concrete lying areas, a 4.0 x 17.0 m straw bedded area and a total of 183.9 m^2^ of slatted flooring. These sows farrow (give birth) in farrowing pens with dimensions of 2.1 x 2.0 m. Each farrowing pen was equipped with a hinge-sided farrowing crate that initially confined the sow immediately post-partum, but could be opened to provide the sow additional space and freedom of movement as the pigs matured. Piglets born to these sows are weaned to ~0.4 m^2^ /animal in groups of 18 piglets. At ~3.5 months of age, the replacement gilts are housed in pens of four gilts at ~0.7 m^2^ /animal until the age of ~6.5 months when they were selected to enter the breeding herd. For the present study, 25 replacement gilts (PIC 1050, Landrace-Yorkshire crossbreed) were placed in behavioral experiments at ages 5 weeks, after first farrowing (10 to 17 months) and during second gestation cycle (11 to 18 months). Each animal was trained and tested for judgement bias approximately two weeks after mixing into large gestational sow pen as primiparous sows. All procedures were performed in accordance to the approved guidelines by the University of Pennsylvania’s Institutional Animal Care and Use Committee, protocol #804656. Data collection methods for the restraint test, response to litter restraint test, and, behaviors recorded at pen mixing have previously been described in our study of the ontogeny of personality trait assessment in production sows [38]. These particular tests were intentionally designed to incorporate routine animal husbandry procedures in order to adapt for on-farm application and to elicit high-arousal and unpredictable situations. A timeline of data collection schedule is summarized in Fig 1.

### Data collection

#### Restraint test

At 5 weeks of age, each piglet (n = 25) was evaluated for behavioral response to being held in the arms of an experimenter for 30 s. This experiment was intended to mimic the routine handling procedures on the farm, such as during piglet processing. Researchers use similar restraint tests (i.e. back-test) as a non-social measure of stress response in order to classify piglets as ‘high-resistant/proactive’ or ‘low resistant/reactive’ based on pre-determined cut points in the behaviors that are measured. The same experimenter performed the restraint test for all piglets. This experimenter stood in one corner of the pen for 60 s to allow for the piglets to acclimate to the presence of a human. The piglets were tested in a semi-random order, with the experimenter bending down to pick up one piglet at a time, often choosing the piglet that is in closest proximity in order to reduce the movement of the piglets in the pen. The experimenter lifted each piglet by a back leg, while supporting the piglet’s chest with their other hand. The test began once the experimenter was standing still while supporting the piglet’s body with both hands, with one hand cradling the torso and the other hand supporting the hips, and resting a side of the piglet body on the human’s chest. The experimenter held the piglet in a prone position firmly and did not move their hands or change the piglet’s position during the 30 s experiment. Each piglet was given a score based on the following scale: 1: No vocal response or physical movement; 2: Less than 5 seconds of soft vocalizations (grunts, squeaks) and/or physical thrashing; 3: 5 to 10 seconds of squealing, grunting and/or physical thrashing; 4: 10 to 20 seconds of high-arousal vocal response (squealing) and/or physical thrashing; 5: More than 20 seconds of high-arousal vocal response (squealing) and/or physical thrashing. The primary experimenter holding the piglet counted in their head continuously (e.g., “one-one-thousand, two-one-thousand, etc.) only when the piglet vocalized or struggled in their arms. A secondary experimenter observing the test recorded the duration of physical struggling and vocalizations continuously using a stopwatch. The scores of each piglet (1–5) were compared between the two experimenters in order to assess the inter-observer reliability (Cohen’s kappa > 0.85). Each piglet was returned to the pen after receiving a single mark on their back using a non-toxic livestock marker to indicate that the animal had been tested.

#### Response during litter restraint test

Each sow’s reaction toward a human handling her first litter during piglet processing was recorded 24 h post-farrowing (sow age range = 10 to 17 months) The piglet processing procedure involved each piglet being removed from the farrowing pen and being placed in a 1.4 x 0.5 x 0.9 m transport cart with straw bedding (QC Supply; Schuyler, NE, USA) to receive an intramuscular injection of iron dextran. The sow was kept in a closed farrowing crate (0.6 x 1.7 m) while the piglets were removed. The test began when the first piglet was removed from the pen and placed in the transport cart, and the test was complete after the last piglet was returned to the pen and the human exited the farrowing pen, with a test duration range of 5.5 to 8.7 min (median = 6 min). Each sow was given a score based on the behaviors and vocalizations she exhibited throughout the piglet processing (Table 1).

**Table 1:** Operational definition of each score given to sows during the litter handle test when their piglets were removed from pen during processing.

Score	Operational Definition
1	Sow does not orient to human, does not vocalize, and remains recumbent during test.
2	Sow takes more than 5 s to change posture while remaining recumbent to orient toward human, and produces less than 5 soft vocalizations (grunts).
3	Sow takes more than 5 s to change posture from recumbent to sitting posture, orients toward human, and produces more than 5 soft vocalizations (grunts).
4	Sow takes more than 5 s to change posture from recumbent to standing and produces mixture of soft and loud vocalizations (grunts and barks).
5	Sow takes less than 5 s to change posture from recumbent to standing, produces loud vocalizations (barks), and attempts to bite or lunge at human in the farrowing pen.

#### Social interaction in gestation pen

The primiparous sows were housed in standard breeding stalls (2.0 x 0.6 m) for seven days after weaning (sow age = 11 to 18 months). Each primiparous sow was then moved from the breeding stalls to the dynamic sow pen, containing 120 to 130 multiparous sows, in batches of 8 to 10 sows approximately 4 to 5 days post-breeding. Each sow was filmed for 1 hour following introduction to gestation pen given that previous studies have reported most aggressive encounters occur at the beginning of group formation [39,40].

Video data was coded for the frequency, duration, and outcomes (displace other or retreat) of fights at mixing of focal sows into the group pen. The duration of fighting was recorded as the start and stop of parallel-pressing; defined as the sows standing side-by-side and pushing the shoulders against each other, with more than three bites initiated or received by the focal sow. Additional behaviors coded included the frequency of aggressive behaviors (bites, chasing) initiated and received and the number of nose-other investigations initiated (Table 2). Video data were analyzed using a continuous focal animal behavior sampling method (The Observer XT 11; Noldus Information Technology, Wageningen, The Netherlands). Video data were coded by three observers, with high inter-observer reliability across all behaviors (Intraclass correlation coefficient > 0.8).

**Table 2:** Ethogram of behaviors coded during the 1-hour post-introduction to an unfamiliar, dynamic gestation pen 5 days following weaning and breeding.

Behavior Event	Operational Definition
Bite	Sows uses teeth to clamp down or scrape another sow’s hear or body.
Chase	Sow purses another sow at a fast pace, trying to reduce the distance between the dyad.
Displace	Sow forces another sow to move away from current location. May or may not be at the end of a fight.
Fight	A dyad of sows engages in reciprocal hits and bites while pressing shoulders against each other (face-to-face).
Hit	Sow uses snout or side of head to hit another sow’s face or body with force.
Lie down	Sow lies lateral or sternal recumbent position with eyes open or closed and not nosing the environment or another sow.
Nose other	Sow places snout near (<4 cm) the snout, head, body or limbs or another sow.
Retreat	Sow moves away from another sow, at a walking or running pace. May or may not be at the end of a fight.

#### Physical measures of welfare

Lesion, lameness, and body condition scores were assessed 3 days post-mixing. Lesion scores were evaluated using a previously designed scale for sows [37]. This scale identified lesion quality (A: Thin (<2mm wide) and shallow; epidermis may be broken with mild surface inflammation; B: Moderately deep cuts (2 to 4mm wide); epidermal and dermal layers may be broken with moderate inflammation; C: Occurrence of wide (>4mm) or deep cuts; dermal layers broken with severe inflammation), and lesion quantity (1:≤5 cuts; 2: 6 to 10 cuts; 3: 11 to 20 cuts; 4: 21 to 30 cuts; 5: ≥31 cuts). Each sow received a quality and a quantity score for the following body regions: anterior (cranial to the caudal aspect of the shoulder), side (between the caudal shoulder and cranial hip), and, posterior (from the cranial hip caudally). For analysis, each quality score was transformed in to a number (A = 1, B = 2, C = 3) and was multiplied by the quantity score (1 to 5). Therefore, the minimum score for each region was 1, while the maximum score for each region was 15.

Lameness scoring was based on the Zinpro Feet First ® (Eden Prairie, MN, USA) scale [41] of 0: sow moves easily with little inducement with no signs of discomfort; 1: sow moves relatively easy, but visible signs of lameness are apparent in at least one leg; 2: lameness is involved in one or more limbs, and the sow exhibits compensatory behaviors such as dipping her head or arching her back; and 3: there is a real reluctance to walk and bear weight on one or more legs, and, it is difficult to move her from place to place on the farm. Body condition scoring was based on the industry standard [42] evaluation of back-fat thickness and prominence of hipbones and spine using the following scale: 1: emaciated; 2: thin; 3: ideal; 4: fat; and 5: overly fat.

#### Judgement bias testing

**Fig 2:**
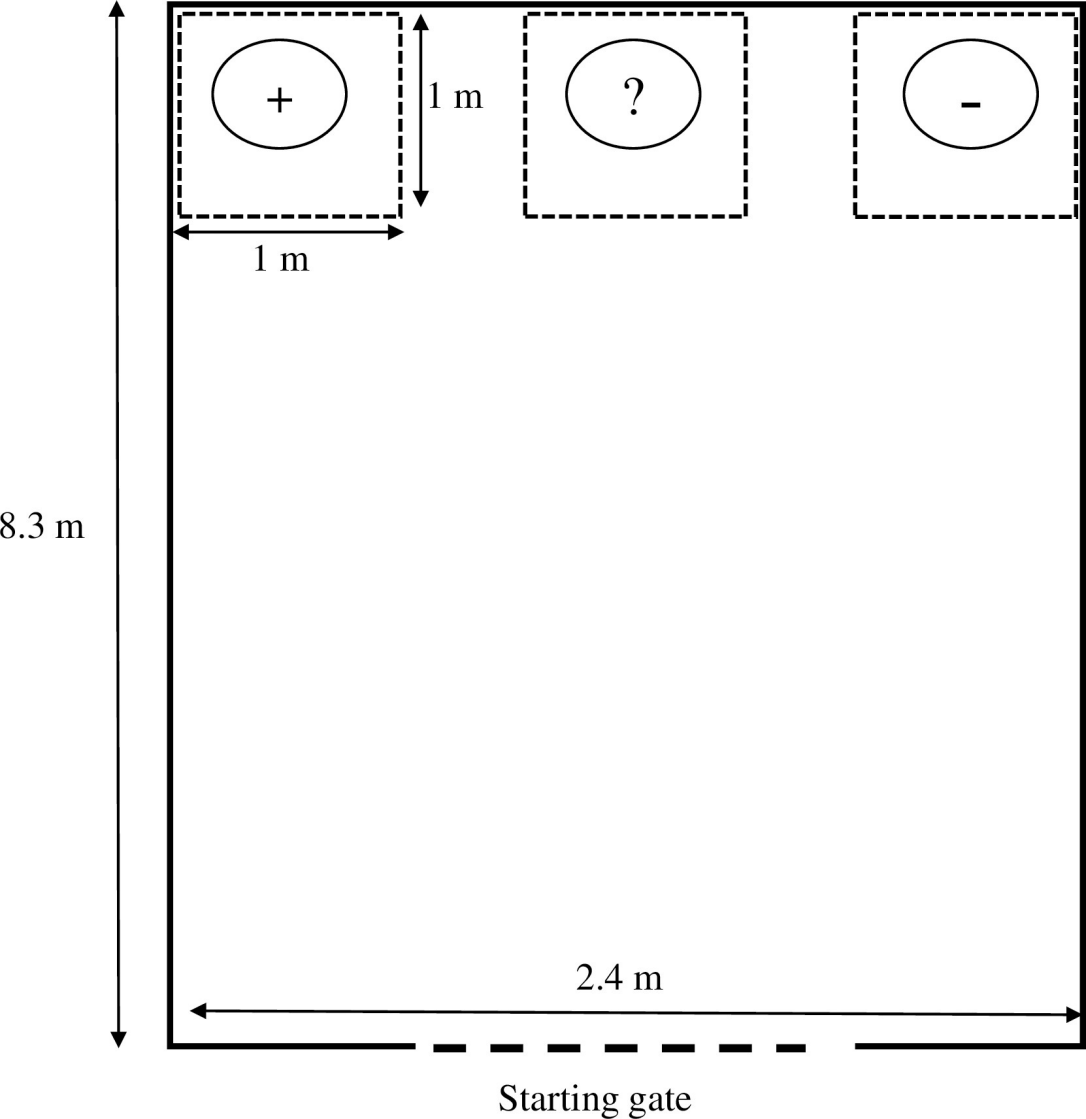
Schematic representation of judgement bias experiment. Sow was held behind gate at starting line (long dashed line). During training, the conditioned feed bowls were place in either far left or right location of the arena. Only one feed bowl was present at a time. Testing trials placed feed bowl in the ambiguous stimulus position (?), equidistant from rewarded (+) and punished (-) locations. Approach was defined as the latency for the sow to leave the starting gate and cross the goal line (short dashed line) with at least one front limb.

Each sow was trained and tested for a judgement bias 2 weeks post-introduction to the large gestation pen. A simplified “go/no-go” task with a single ambiguous cue was designed to facilitate training and throughput to ensure that we were able to test this cohort of animals at a similar age and stage of gestation. Experimental set-up, training and testing of the behavioral task was previously described in our study of judgement bias assessment in a separate cohort of multiparous sows [29]. For both the positive and negative stimuli, a single 25.4 cm in diameter metal pet food bowl was filled with 200 g of corn-soy meal and placed in the far left or right corner of a 2.4 x 8.3 m experimental field (Fig 2). The first 3.7 m of this field was walled with steel gating, while the remainder of the field was walled with cement. The designation of positive versus negative stimulus location (left or right) was randomized for each sow. Using operant conditioning of paired associations, sows were trained to distinguish between the two visual stimuli based on the location of the food bowl. Only one bowl was present in the field during each trial, with a primary researcher standing behind the bowl, holding the red flag behind their back, in order to keep a consistent protocol. This primary researcher kept their gaze forward and did not interact with the sow. A second researcher acted as the animal wrangler; releasing the animal from the starting gate and retrieving the animal from the experimental area after each trial. Animals were trained at approximately the same time each day (1000–1400) for a maximum 12 trials or a maximum time of 30 minutes. The order in which the animals were trained and tested was dependent upon the time of their last meal, in order to ensure that each animal was trained at least 4 hours after eating their daily feed at the ESF stations.

On the first day of training, the sows were conditioned to approach the bowl in the positive location over a series of ten trials. Approach was defined as the latency for the sow to leave the starting gate and cross the goal line of a 1 x 1 m area containing the feed bowl with at least one front limb. The distance from the starting gate to the goal line was 7.3 m. If the sow approached the positive stimulus, she was allowed to consume the corn-soy feed for 10 s. The second day of training began with a positive trial for reference, followed by a minimum of 4 negative trials, and ending with one positive trial. If the sow approached the feed bowl in the negative stimulus location, the standing researcher would wave a red flag over the bowl for 20 s, preventing the sow from consuming the feed. The third and fourth training day began and ended with a positive trial, with eight randomized positive or negative trials in between. Sows were considered trained if they approached the positive stimulus within 20 s for 100% of the positive trials and if they did not approach the negative stimulus at least 50 s for at least 80% of the negative trials. The speed criteria were chosen based on average walking speed to approach bowl from starting gate for a sow. The minimal successful no-go response was determined empirically based on observation of the sows and distribution of the data [35]. The experiment day included three trials: a positive trial, a negative trial, and an ambiguous trial. In this ambiguous trial, the feed bowl was placed in the middle of the experimental field, equidistant to the positive and negative stimuli (Fig 2). The latency of approach to this ambiguous stimulus was recorded, with a maximum time of 60 s (i.e., no-go). Additional probe cues, placed between the conditioned stimuli and the ambiguous bowl position, were not used in this experiment as the pregnant sows were unable to perform multiple trials within a 30-minute time frame. Given the physical limitations of the animals, and that an elongated testing period to include probe cues would find the sows in heavier body conditions due to growing gestation, we relied on behavioral response to a single ambiguous cue.

### Data analysis

#### Principal component analysis

The eleven behaviors recorded during pen introduction were transformed into composite traits using principal component analysis (PCA) with a Varimax rotation (IBM SPSS Statistics 24, Armonk, NY, USA). Prior to PCA, the adequacy of the data was examined, ensuring that Bartlett’s test of sphericity was significant (P<0.001) and the Kaiser-Meyer-Olkin measure was high (KMO = 0.64). Components which had eigenvalues greater than 1 were retained for interpretation. Behaviors were grouped based on a loading cutoff of 0.6. Sows received a single component score for each trait using the least squares regression approach. Significant relationships among the variables were assessed using a non-parametric Spearman’s rank-order correlation (P<0.05).

#### Modeling of study outcomes

A linear mixed-effects model with restricted maximum likelihood was implemented in R using the lme4 package [43]. We analyzed individual animal single trial latencies as a continuous outcome variable, with latency being defined as the time to approach the specific stimulus cue. The initial model included as fixed effects: probe location, behavioral trait scores from the principal component analysis of social interaction, skin lesions scores, restraint test scores, and response to litter restraint test, as well as their interactions with probe location. Fixed effects were dropped if they did not influence model fit as assessed using ANOVA. Lesions scores and the submissive and active trait scores were dropped from the final models for this reason. Sow identity was included as a random effect. To better understand interactions in the models, post hoc linear models were fitted to examine how the different variables above influence the time to approach specific probe locations (positive, negative and ambiguous). Visual inspection of residual plots did not reveal any obvious deviations from homoscedasticity or normality. P-values were obtained by likelihood ratio tests of the full model with the effect in question against the model without the effect in question. Ordinal variables such as lesions scores and human interactions scores were only included in models as independent variables [44]. In these cases, model coefficients can be more difficult to interpret and as such are not presented. Median and interquartile range (IQR) were used to describe these count data whereas mean and standard error of the mean were employed for latency measures. Power analysis using power.f2.test in R revealed that with the study sample size, a significance of 0.05, and a power of 0.8 that we could detect an effect size of 0.35 or larger.

## Results

### PCA analysis

Three principal components which explained 79.2% of the variance were extracted from the dataset (Table 3). The first principal component had strong (>0.6) positive loadings for the following behaviors: number of bites and chases initiated, the duration of chasing another sow, the number of displacements of other sows, and the total number of fights engaged. The second principal component had strong positive loadings for the following behaviors: number of retreats from another sow, the number of bites received, and the duration of being chased by another sow. The final principal component had a strong positive loading for the number of nose-investigations of other sows, while it had a strong negative loading for the duration of lying down during pen mixing. Based on the behaviors which loaded onto each component, they were labeled: *aggressive*, *submissive*, and *active/exploratory*.

**Table 3:** Obliquely rotated principal component loadings for behaviors recorded during the 1-hour post-introduction to an unfamiliar, dynamic pen as primiparous sows.

	PC1	PC2	PC3
	Aggressive	Submissive	Active/Exploratory
Bite–initiate (#)	**0.92**	0.07	0.14
Chase—initiate (#)	**0.87**	-0.02	0.12
Chase–initiate (s)	**0.80**	-0.09	0.001
Displace (#)	**0.72**	-0.20	0.54
Fight (#)	**0.86**	0.15	0.14
Bite–receive (#)	0.58	**0.65**	-0.06
Chase—receive (#)	0.20	**0.92**	0.07
Retreat (#)	0.41	**0.78**	0.04
Lie down (s)	0.12	-0.39	**-0.78**
Nose other (#)	0.42	-0.19	**0.73**
Eigenvalue	4.3	2.1	1.5
Variance explained (%)	42.9	21.4	14.9

Rotation Method: Varimax with Kaiser Normalization. Rotation converged in 5 iterations.

Component loadings >0.6 (bold face) were clustered to create composite traits.

### Judgement bias assessment

**Fig 3:**
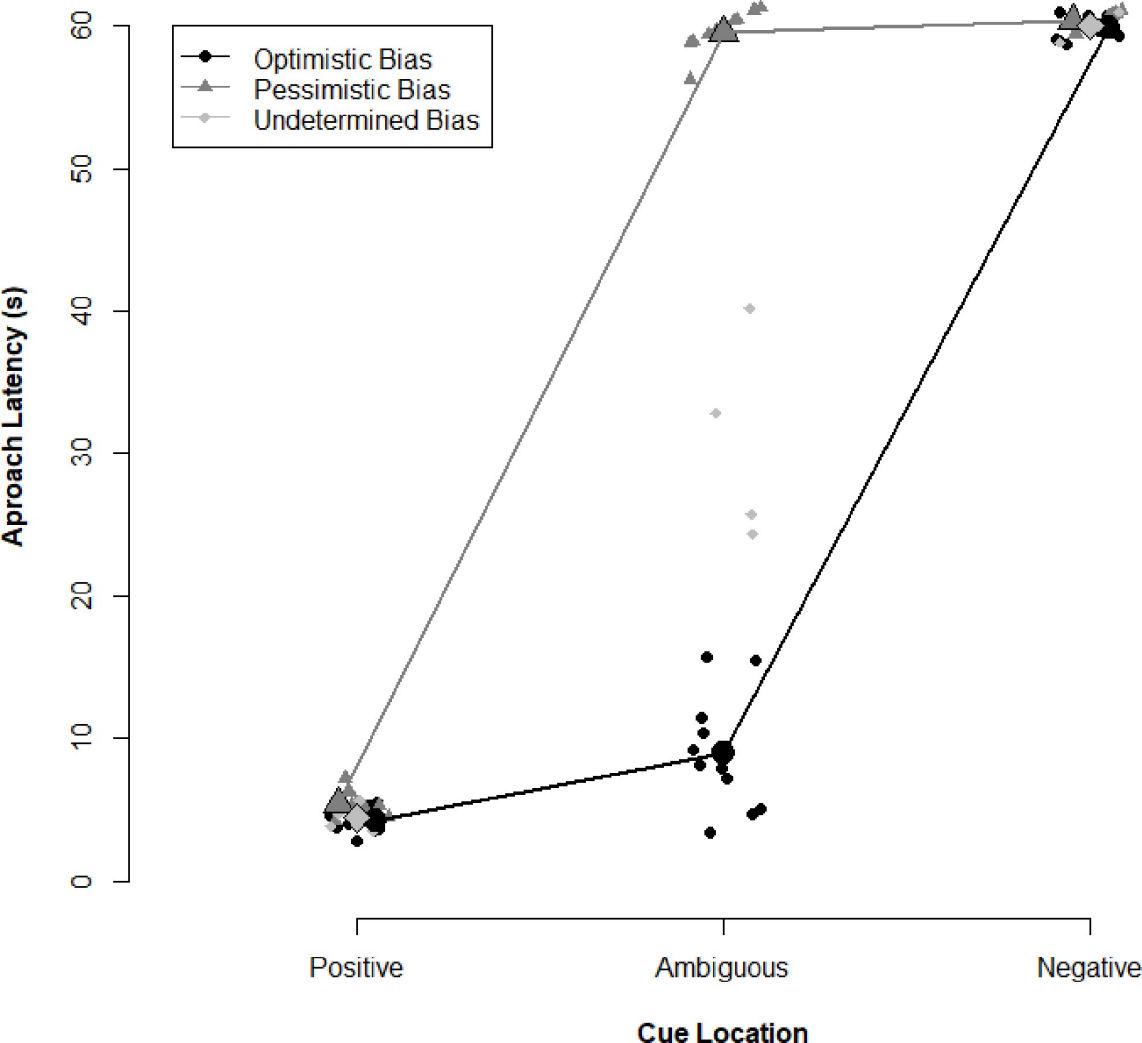
Judgement bias outcomes vary across group housed sows. Individual latencies for sows to approach the positive, ambiguous, and negative stimuli area displayed as well as means for animal exhibiting either an optimistic bias (ambiguous approach < 20 s), an undetermined bias (ambiguous approach, between 20-50s) or a pessimistic bias (ambiguous approach > 50 s). Both optimistic and pessimistic bias animals approach the positive and negative conditioned stimulus with similar latencies independent of their judgement bias.

Of the 25 sows trained, only one sow did not meet the task requirements on test day and was removed from the analysis. The remaining 24 sows approached the positive cue in less than 20 sec and failed to approach the negative cue for at least 50 sec. When approaching the ambiguous cue, eleven animals exhibited a latency of less than 20 s with an average time of 9.0 +/- 1.2 sec; nine animals took longer than 50 s with an average time of 59.6 +/- 0.4 s; and 4 animals exhibited a latency of between 20 and 50 s with an average of 30.8 +/- 3.8 s (Fig 3).

### Relationship between traits and judgement bias

**Fig 4:**
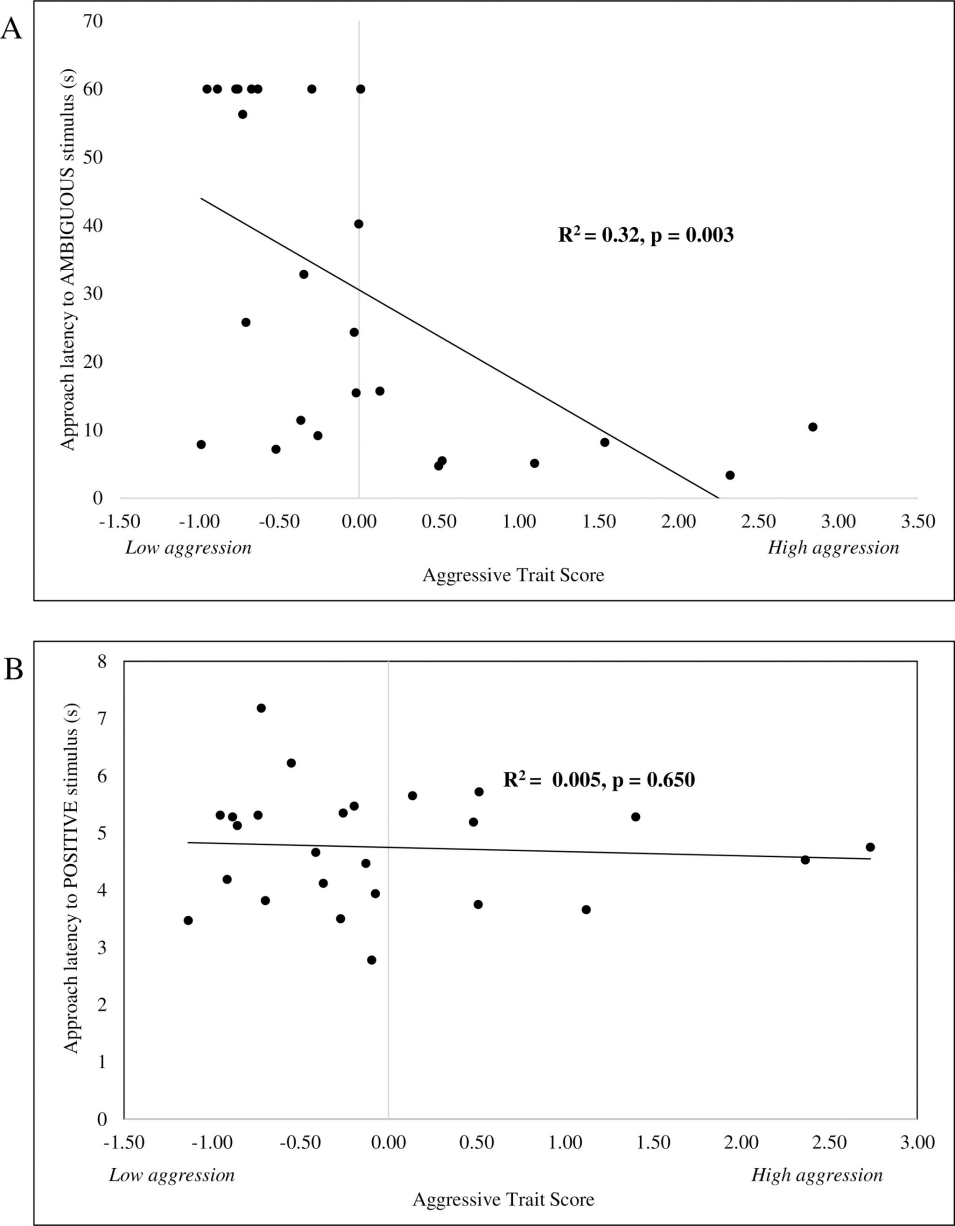
Personality influences judgement bias of gestating sows. (A) Significant relationship between the trait scores for aggressive/dominant and the latency to approach the ambiguous cue (p = 0.003), with more aggressive/dominant sows approaching the ambiguous cue faster; indicative of a positive judgement bias. (B) Aggressive/dominant animals, however, do not approach the positive cue faster than less aggressive animals (p = 0.65).

A linear mixed effects model revealed that both the location of the stimulus (χ^2^ (2) = 102.83, p<0.001) and trait scores for *aggression* (χ^2^ (1) = 8.81, p = 0.003), restraint test score (χ^2^ (1) = 5.44, p = 0.020), and the response to litter restraint (χ^2^ (1) = 4.93 p = 0.027) significantly affected the latency to approach ambiguous stimulus. There were no significant effects of the *submissive* or *active/exploratory* trait scores on the latency to approach positive, ambiguous or negative stimulus. Interactions between probe location and each of these three variables were also significant (*aggressive* trait score: χ^2^ (2) = 21.40, p < 0.001; restraint score: χ^2^ (2) = 11.24, p = 0.004; and litter restraint score: χ^2^ (2) = 11.27, p = 0.004). Specifically, the approach to the ambiguous stimulus was affected by the trait scores for *aggressive* (F(1,22) = 11.49, p = 0.003), with more aggressive sows displaying shortened approach times to the ambiguous cues or a more optimistic judgement bias (Fig 4A). However, aggressive sows were not quicker in general as there was no significant relationship between the approach to the positive cue and the aggressive trait score (F(1,22) = 0.21, p = 0.648; Fig 4B). Similarly, the approach to the ambiguous stimulus also was affected by the restraint test score (F(1,22) = 5.74, p = 0.026), with sows that exhibited more resistance to restraint as a piglet displaying more optimistic judgement bias. These sows also were not quicker in general as there was no significant relationship between the approach to the positive cue and the restraint test score (F(1,22) = 1.57, p = 0.223). The approach to the ambiguous stimulus also was affected by the response to litter restraint scores (F(1,22) = 5.55, p = 0.028), with sows that exhibited a stronger response to the restraint of their litter displayed a more optimistic judgement bias. These sows also were not quicker in general as there was no significant relationship between the approach to the positive cue and the response to restraint litter (F(1,22) = 0.00, p = 0.951).

### Physical measures of welfare

**Fig 5:**
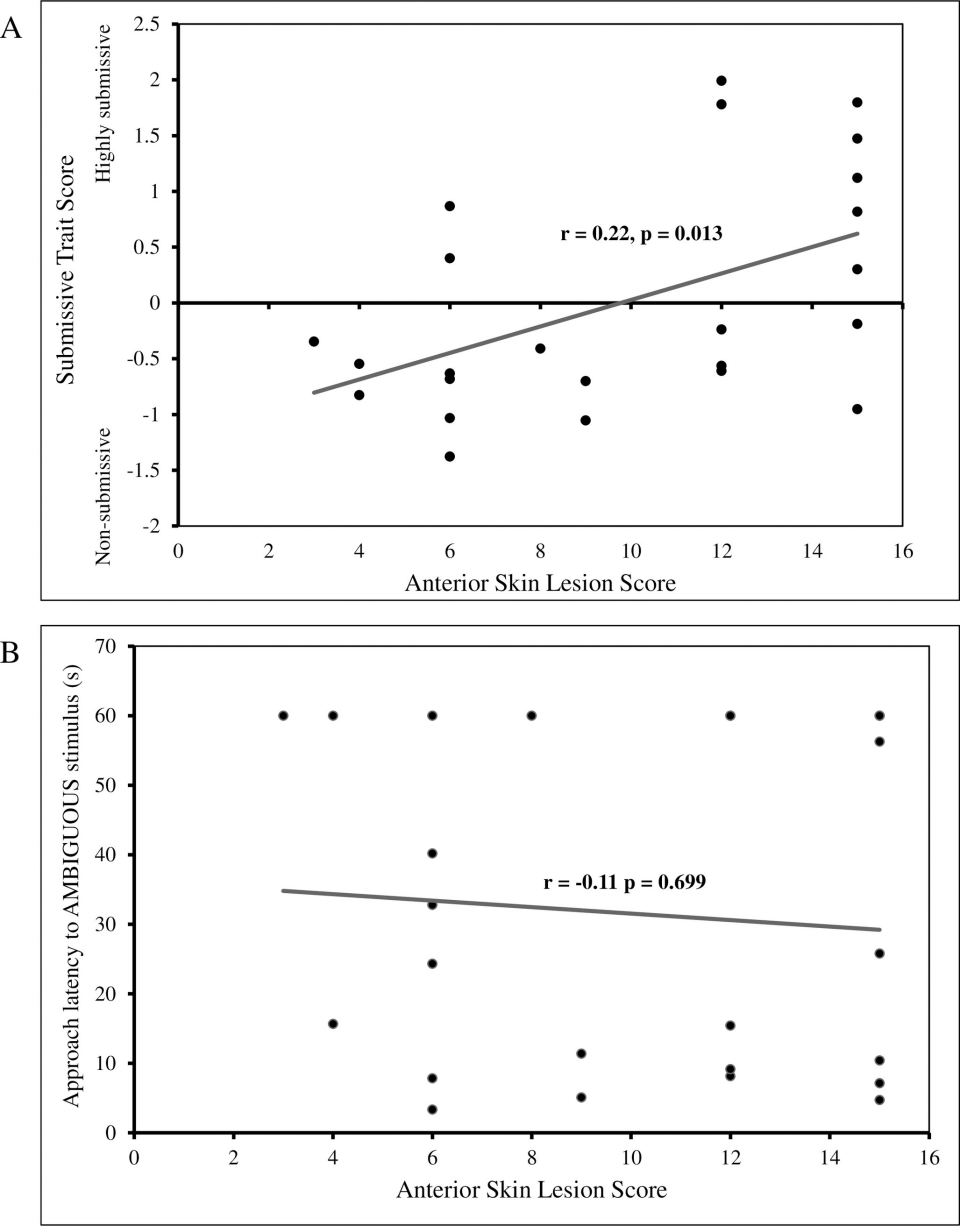
Physical measures of animal welfare do not predict with judgement bias of gestating sows. (A) Significant relationship between anterior skin lesion scores and submissive trait scores (p = 0.013) as animals with more severe skin lesions had higher submissive trait scores. (B) Animals with less severe anterior lesions scores, and thus better physical welfare, did not exhibit faster approaches to the ambiguous cue as no significant relationship was observed between lesion scores and latency to approach the ambiguous cue (p = 0.699).

Skin lesions acquired following the introduction of study animals into an unfamiliar group of sows ranged from a score of 1 to 15 and were most common in the anterior region (median = 12; IQR = 9), intermediate for side lesions (median = 6; IQR = 5), and least common in the posterior region (median = 4; IQR = 2). A significantly positive relationship was observed between the *submissive* trait scores and lesion scores of anterior region 3 days post-mixing (F(1,22) = 7.24, p = 0.013) (Fig 5A). However, no significant relationship was observed between any of the other regional lesion scores and the *submissive trait* or any other lesion scores and the other two personality traits (*aggressive/dominant or active/exploratory)*. There also was no significant relationship between the lesions scores of each body region and judgement bias (Fig 5B). The latency to the ambiguous cue was not significantly related to an animal’s anterior lesion score (F(1,22) = 1.54, p = 0.699), side lesion score (F(1,22) = 1.05, p = 0.316), nor posterior lesions score (F(1,22) = 0.03, p = 0.861). Furthermore, neither lameness nor body condition score impacted judgement bias. No animals were evaluated to be lame during the study (i.e., a lameness score ≥ 2), and thus, lameness could not account for pessimistic biases observed in nine sows. Similarly, only 3 of the 24 of the animals at the time of judgement bias testing had a sub-normal body condition (BCS = 2) and the latency to the ambiguous cue was not significantly related to an animal’s body condition score (F(1,22) = 0.67, p = 0.423).

## Discussion

Optimistic, pessimistic, and undetermined judgement bias were observed among a cohort of 25 out of 130 gestating sows that shared similar genetics and rearing conditions, and lived in the same pen. We did not find that animals with increased lesion severity displayed more pessimistic judgement biases. For this study cohort, variation in body condition and lameness was too small for meaningful analyses in regards to performance in judgment bias test. Instead, head and shoulder lesion severity following introduction to the gestation pen was significantly correlated to the personality trait of *submissive*. In our previous work, sows which had higher *aggressive* trait scores were evaluated to have more severe lesions in the anterior region [37]. This was not found in the present study and may be the result of a smaller sample size, but it does indicate that the variability of lesions acquired for group housed sows is linked to the trait of *submissive/non-aggressive* and *dominant/aggressive*. Previous reports have also found that higher lesions scores are not necessarily indicative of a more aggressive sow, but instead, may signify something more about the aggression level of the pen [45]. Furthermore, we found that sows which were determined to be more resistant during restraint as a piglet, more aggressive toward humans as an adult when their piglets were handled, and more aggressive to conspecifics during social mixing, were more likely to display optimistic biases as compared to non-responsive gilts and non-aggressive sows. Taken together, these results suggest that the judgement bias paradigm used in the present study more likely was evaluating a sow’s response to risk as it relates to consistent individual differences in behavior (i.e., personality) and raises the question of how personality attributes contribute to the subjective mood state (i.e., mental welfare) of the sow. Further research is needed to evaluate if this spatial judgment bias task is capturing emotional differences in temporary appraisal bias (i.e. mood state) or a more permanent factor of the animal’s character (i.e., affective or coping style) [46].

The spatial learning task has previously been used to assess judgement bias in younger pigs based on variation in housing enrichment [13], following social isolation [47], and following a change to the stocking density [30]. Unfortunately, the use of such cognitive methods to evaluate affective states of mature swine, like breeding sows and boars, is not as well-established in the literature. These breeder animals live the longest lives of any animals on a swine farm and have the potential to endure the most suffering from negative affective states. Current welfare assessments for breeding sows focus on physical ailments (e.g., lameness, body condition and skin lesions), as well as being the target of aggressive behaviors (e.g., bites, scratches, displacement). However, given that recent work in dairy heifer welfare has reported a lack of correlation between behavioral indicators of anhedonia and the number of agonistic interactions received during regrouping [48], there is a need to carefully critique our assumptions of how welfare is experienced by the animal. If we are using judgement bias testing as an indicator of subjective mental welfare of animals, we also need to better understand how, and if, the degree and duration of physical comprise contribute to an animal’s psychological welfare state.

Previous research evaluating comprehensive welfare assessments of stalled versus free-ranging horses found that individuals which live in more restrictive, non-social environments not only displayed the highest levels of behavioral and chronic health-related problems, but they also showed more hesitation to ambiguous probes (i.e., negative judgment biases) [49]. In our study, however, there was not a significant difference in the location, quantity, or quality of lesions between sows with optimistic biases and those with pessimistic biases. An *a prioiri* assumption would be that sows with more severe lesions would display more pessimistic biases; given that the lesions could result in a negative state like pain (or be caused by engaging in negative social behaviors and result in a fear or anxiety state). This relationship, however, was not found, and thus our data do not support an impact of physical measurements of welfare (i.e., skin lesions, body condition) on judgement bias in sows. Given the two-week delay between the acquisition of lesions at mixing and the judgement bias testing, the time horizon over which these lesions could negatively impact the sow’s mood from pain, fear or anxiety maybe relatively short-lived compared to the several weeks that it takes for the skin lesions to heal [50].

In behavioral ecology, the relationship between speed-accuracy tradeoffs and personality differences in risk-reward tradeoffs are examined as impacting individual fitness through fast or slow response to environmental change [51,52]. Similar to the *bold-aggressive* behavioral syndrome, which is represented by fast-responding animals that take large risks for high reward (with poor accuracy in gaining reward), animals with *proactive* coping styles are also bold (e.g., shorter approach/attack latencies), aggressive, and rely on routine behavior (i.e., low flexibility in response to change) to cope with challenges in their environment [53]. Reactive individuals are more cautious (e.g., longer approach/attack latencies), less aggressive and are more adaptive to change by developing different behavioral patterns. In keeping with this theme, we have previously reported that dominant sows exhibit shorter latencies to approach an ambiguous stimulus [29]. In order to improve upon the methods conducted in this study, it is recommended that sows are placed in multiple contexts (e.g., appetitive-consummatory behavior, social behavior, human interaction) and each test is repeated to establish trait consistency.

Several possible limitations to our study warrant discussion. One potential limitation of this study is that we used a locomotion-based go/no-go task, which prevented severely lame sows from participating in the trials. It is thus possible that more negative states of physical welfare, such as resulting from more severe lameness, might have a greater impact on the sow’s subjective mental experience, and thus, influence judgement bias. Another possible limitation of this study was that it was only sufficiently powered to detect large effect sizes. Thus, the failure of this study to find a relationship between physical welfare and judgement bias could also be explained by there being only a small contribution of physical measures of welfare to affective state as such we would not have observed it. Another possible limitation of this study is that only one ambiguous probe cue was used in the go/no-go paradigm, instead of a more common three intermediate probe cues [8,9]. The operant conditioning schedule for this study was intentionally limited to one week in order to reduce the chance for fatigue or physiological factors related to pregnancy to influence behavior in the locomotion task. Each sow was trained for a maximum of 30 minutes per day, as previous research reports that gestating sow spend over 70% of their day lying down in rest or sleep state. In addition, commercial breeding sows are fed approximately 30% of ad-libitum consumption per day, which meets nutritional needs, but does not result in satiety [54]. Therefore, our sows were chronically hungry. Given that the reward for our study was access to their soy-corn diet, presenting multiple ambiguous trials would be difficult to interpret, as all sows were approaching a bowl if it was not punished (i.e., an unrewarded bowl was still reinforcing for these hungry sows). Future work could attempt to either pre-feed sows prior to testing and training, but research has shown that approximately 6.8 kg is need for a sow to be sated, or, the reward could be non-food variety, such as access to straw to enrichment. A final possible limitation is in how the data was collected. In the latency to approach the ambiguous cue, the trial was ended if after 60 seconds the sow failed to approach. This data has been described ‘continuous but censored’ and the concern is that these censored latencies might underestimate pessimistic bias in quantitative model [55]. However, the results suggest that the censored data does not influence our interpretation of the findings. In Fig 4A, longer latencies for the animals with pessimistic biases only would have accentuated the inverse relationship between latency and trait score. In Fig 5B, the animals with censored latencies were observed across the full range of lesion scores consistent with no relationship between latency and skin lesion scores.

An additional caveat to interpreting animal behavior in judgement bias tests is that bold, exploratory animals may appear to perform better at the go/no-go task simply because they are quicker to respond. For example, mice which display fast exploration of the open areas in an open field test (i.e., bold/exploratory trait) show superior performance in a variety of spatial and discrimination tasks than non-exploratory or cautious mice [56]. It can be argued that animals which are less prone to novelty seeking, and are slow to explore their environments, may appear to be more hesitant in the go/no-go task; and thus, are assumed to experience more negative affective states than animals which are quick to process and respond to environmental stimuli. This very notion has been reported in a study with laboratory mice, with more exploratory, less neophobic mice taking time to investigate novel, ambiguous arms than reach the reward end [57]. An inherent reason for using judgement bias testing to assess affective states in animals is to investigate how emotions impact an organism’s judgement of risk in their environment (through approach-avoid behavior). However, without overt manipulation of an animal’s subjective mental state (e.g., painful husbandry practices, as seen in negative judgement bias post-horn disbudding in dairy calves [58]), or control for the pleasure versus aversive experience of the reward and punishment (e.g., 6 versus 1 apple slice for grizzly bear judgement bias test [59]), it is difficult to ascertain the reason for an animal to hesitate approaching or responding to an unknown stimulus; it could be caused by a short-term affective state, or a long-term affective style (i.e., personality trait [60]).

## Conclusions

In our longitudinal study of personality development among a cohort of domestic sows, individuals which were determined to be more resistant during restraint as a piglet were more aggressive as an adult during social mixing and when their own piglets were handled during processing [1]. The results from the current study indicate that these aggressive individuals were also more likely to display optimistic biases as compared to non-aggressive, non-responsive sows. This suggests that animal personality may impact cognitive processes, perhaps through different emotional states, and influence a sow’s appraisal of unknown stimuli in the environment. Although the individuals of this study were housed in same enriched environment, both optimistic and pessimistic biases were observed and these judgement bias outcomes did not correlate with contemporaneous physical measures of animal welfare. Based on the results of this study and reconciling a growing body of evidence in the literature, we suggest that individual differences in behavioral traits generate different emotional responses that can contribute to the animal’s overall affective state. This work also supports variable contributions to affective state given the poor correlation of judgement bias with transient physical compromise. Our findings highlight the complexity of how different factors impact an animal’s overall affective state and argues the need for additional, complementary measures in future JBT studies on group-housed gestating sows, including personality assessment.

## Supporting information

S1 Data (XLSX)
