# Supplementary material for: Judgement bias of group housed gestating sows predicted by behavioral traits, but not physical measures of welfare
Source: PLoS One. 2022 Feb 25;17(2):e0264258. doi: 10.1371/journal.pone.0264258 (PMC8880926; doi:10.1371/journal.pone.0264258)
Supplement: Attachment — Submitted filename: PLOS ONE revision note CLEAN.docx [file pone.0264258.s002.docx]

Revision note

PONE-D-21-16018

Judgement bias of group housed gestating sows predicted by personality traits, but not physical measures of welfare

Editor:

When submitting your revision, we need you to address these additional requirements.

1. Please ensure that your manuscript meets PLOS ONE's style requirements, including those for file naming.

**Response: PLOS ONE style requirements were addressed.**

2, We note that the grant information you provided in the ‘Funding Information’ and ‘Financial Disclosure’ sections do not match. When you resubmit, please ensure that you provide the correct grant numbers for the awards you received for your study in the ‘Funding Information’ section.

**Response: This has been addressed during re-submission.**

3. Thank you for stating the following in the Acknowledgments Section of your manuscript:

“The authors would like to thank the summer research interns of the Swine Research and Teaching Center for their assistance in data collection. This work was supported in part by Kraft Foods Oscar Mayer, Pig Improvement Company, Pennsylvania Pork Producers Council, Pennsylvania Soybean Board, Pennsylvania Department of Agriculture, and the American Society for the Prevention of Cruelty to Animals”

We note that you have provided additional information within the Acknowledgements Section that is not currently declared in your Funding Statement. Please note that funding information should not appear in the Acknowledgments section or other areas of your manuscript. We will only publish funding information present in the Funding Statement section of the online submission form. Please remove any funding-related text from the manuscript and let us know how you would like to update your Funding Statement. Currently, your Funding Statement reads as follows:

“This work was supported in part by Kraft Foods Oscar Mayer, Pig Improvement Company, Pennsylvania Pork Producers Council, Pennsylvania Soybean Board, Pennsylvania Department of Agriculture, and the American Society for the Prevention of Cruelty to Animals. The funders had no role in study design, data collection and analysis, decision to publish, or preparation of the manuscript”

Please include your amended statements within your cover letter; we will change the online submission form on your behalf.

**Response: We have removed all references to funding support from the acknowledgments and included this information in the funding statement.**

Reviewer #1: This is an important subject, to challenge the recent tendency to over-interpret results from cognitive bias paradigms as de facto evidence of good or bad welfare. It would be nice to see consideration of alternative explanations for cognitive bias results become the norm.

Reviewer #2: This paper aims at exploring what factors influence performance in judgement bias testing in group-housed sows with an emphasis on personality traits and measures of physical aggressions. This is an important area of research given the need for a better understanding of affective states of farmed animals and how it can successfully relate to other welfare measures. I think the study has merits, but I feel it could really be improved. I spent quite some time making both general and detailed comments that I hope will help the authors.

General comments

• The literature considered in this manuscript is rather incomplete as work on other species (dairy calves and rats) has not been integrated. I would strongly recommend including what has been done in other species given that only a few studies have explored individual variation in response to judgment bias. For now, the authors do not really refer to studies exploring this link but rather mention their existence. I think the integration of these studies will provide more context and should help the authors better communicate their results, especially in terms of how moods can be considered both as reflecting emotional states and/or personality differences.

**Response: Previous studies which examine personality, coping style, or behavioral syndrome in a variety of specie, as it relates to risk-taking, appraisal or judgment bias, have been incorporated throughout the introduction and discussion.**

• The rationale behind the research question could be improved. For now, the reader is left with limited information on why the authors did this study and why it is important.

**Response: The last sentence in the second to last paragraph has been revised to clarify why this study was conducted with this particular population of swine.**

• I’d like the authors to discuss more some limitations of their study. For instance, the authors used only one ambiguous cue presented only once, which is at odds with the literature. The authors need to explain why they made that choice and more effectively explain how this could have affected their results (i.e., animals may be more incline to react to ambiguous cues as novel element in their environment on the first exposure vs. on repeated exposures).

**Response: The reasons for only one ambiguous cue (animal fatigue, chronic animal hunger) have been incorporated into the methods. We have addressed the limitation of one ambiguous trial in the discussion with greater details; including ways to improve upon our study design in the future.**

• Many scientists would argue that personality traits need to be assessed over time and contexts. In addition, tests were not all standardized (e.g., the social interaction test was more a regrouping experiment), which is a limitation of the study. I would recommend to keep the concept of personality for the discussion but I may be convinced otherwise if the authors clearly stress the consistency of their outcome variables.

**Response: We agree with this critique. We have replace the word ‘personality traits’ with ‘behavioral traits’ throughout the manuscript, but maintain the theme of individual differences in behavior consistent over time were found with the dataset; as we have established this difference using the same study cohort in another peer reviewed study (Horback and Parsons, 2018, *Behavioral Processes*).**

• What is the point of the JBI? I would remove this part as the authors do not seem to use it for further analyses.

**Response: The Judgment Bias Index was created to control for differences in the approach speed between sows (i.e., some animals simply always moved slower than others). However, in this study there was not that much difference in locomotion rates between sows and hence why as the reviewers point out, this index is not used for further analysis or interpretation. As such we have removed all information about the JBI from this manuscript.**

Abstract

L21 states?

**Response: The ‘s’ has been added.**

L22 I feel the authors should give more information to the reader so that it is easier to grasp how JBTs work. I would add elements explaining pessimistic and optimistic responses.

**Response: Sentences have been added to explain the optimistic/pessimistic interpretation of animal behavior in JBTs.**

L26 Maybe better to split this sentence?

**Response: This sentence has been split into two.**

L36 I would tone down the conclusion as the authors did not manipulate affective states in this study.

**Response: This final sentence to the abstract has been revised to reduce the strengths of conclusions to be taken from our data.**

Introduction

“Due to both government legislation and consumer demands, the practice of housing of gestating gilts and sows as individuals in stalls has been banned in many developed countries, including the EU1, Australia, New Zealand, Canada, South Africa and ten states in the United States2,3”

This sentence could be read as stalls are forbidden in these countries. In practice animals can still be confined during the lactation period and sometimes for a few weeks during the gestation period. Can the authors add clarification here?

**Response: This sentence has been revised.**

“The personality of an organism refers to the relatively consistent behavioral reactions of an organism across contexts and is most clearly expressed in novel and unpredictable situations17”

This sentence is not super clear + avoid repetitions (maybe replace organism by individuals or animals). Overall the authors do not clearly articulate how their measures can be used to reflect personality traits in sows.

**Response: We have included a more thorough definition of personality and included a segue sentence to describe how tests to evaluate individual differences in behavior should be novel or unpredictable to reveal high-arousal responses.**

“and the expression of pain21” Again there is more recent work that would better fit your article as variation in jbt responses has been linked to pain in calves (see Lecorps et al., 2020).

**Response: This source has been incorporated to the introduction.**

L70 to 74: I would push the authors to also consider the work being done in rats and dairy calves.

**Response: Given that there is work done relating personality to performance in JBTs for many other species (dolphins, mice, rats, dairy cows, etc.), we have decided to expand on this relationship in the discussion, rather than elongate the introduction. This sentence does state that the following studies are examples of this relationship.**

L75 the logic needs some work in this paragraph. The authors start by saying that there is not enough research being done on affective states (from this paragraph it feels like the authors say that research on affective states in pigs are limited to judgment bias studies). Then they say that there is not enough research being done on personality traits. However, they do not really stress out why we need to explore the relationship between response to jbt (hypothesized to reflect on mood) and personality traits. Why is this important? One or two additional sentences would help the reader following the point made by the authors. Overall, I think this is one of the points I’d like the authors to focus on. The conceptualization of the study and rationale for the question asked is not very clear in the introduction. The paper would be much stronger if these points were improved.

**Response: This paragraph has been revised to include research on assessing swine affective states other than JBTs, as well as to highlight the lack of information on breeding sow personality (instead of piglet personality). Sentences were added to clarify the need for more information on if/how sow personality influences performance in JBT (as little is known about this breeding population).**

L95 will impact response to judgment bias tests

**Response: This change has been made.**

Methods

L158 a dot is missing

Personality tests used seem different from the rest of the literature. Can the authors comment on that? What is typically done? Why not mimicking what Asher et al 2016 (one of the key reference for this work) did? I like the fact that the procedures intended to use routine procedures happening to piglets/sows in commercial settings, but I think this choice needs to be justified and explained.

**Response: A sentence has been added to explicitly state and defend the use of these tests which can be incorporated into routine animal husbandry, for a more generalizable and practical use. It should also be noted that the study population is different between our study and Asher et al. In this manuscript, we follow animals from 5 weeks of age to just beyond conception of their second litter (a 50 week or more time span) whereas Asher et al focused on grower pigs that varied in age over a 6 week period from 4 to 10 weeks of age. The personality test that are used here are previously described in the following publications (Horback and Parsons, 2016 & 2018).**

L171 An illustration of my doubts regarding the first paragraph of the introduction. The readers may be led to think that the ban of stalls/crates means animals are no longer kept in confinement during their life, which is not the case. However, this still happens at various times.

**Response: The first paragraph of the introduction has been modified to reduce the insinuation that sows are never in stalls or crates.**

L205 “sow moves easily with little inducement. She is comfortable on all her feet” I would link these two sentences, otherwise it is a little confusing. Same for the following sentences. Another option would be to have a table for this.

**Response: The description of the lameness scale was revised to have one sentence per score to reduce confusion.**

“Sows were considered trained if they approached the positive stimulus within 20 s for 100% of the positive trials and if they did not approach the negative stimulus at least 50 s for at least 80% of the negative trials.”

Can the authors explain why they chose these criteria? Do they come from previous studies?

**Response: These criteria are based on average walking speed of the sows and a clear break in the performance of the sows looking at the data (explained in Horback & Parsons, 2018, Behavioral Processes). This explanation has been added.**

L254 Why only one ambiguous trial? This is very unusual as most JBT studies would use at least multiple exposure to the same ambiguous cue or to multiple cues. How confident are the authors that this was enough? I feel this needs to be justified.

**Response: We understand this concern as all other spatial judgement bias tasks include probe cues in the assessment. We have included in the methods that our pregnant sows were not able to be placed in multiple trials, and thus additional probe cues, as they became physically exhausted from standing and walking after 30 minutes. Not described in the manuscript, but for the reviewers’ knowledge, we have developed a non-strenuous activity (joystick movement from stationary/sitting animal) to allow for probe cues to be used in future judgment bias tasks with breeding sows.**

L259 It is not clear if the authors mean they used all variables collected in all the different tests (except JBT) or just “during pen introduction” (i.e. social interactions).

**Response: This sentence has ben revised to clarify that it is only the behaviors during introduction to pen as adults that were placed in PCA.**

L268 please state here how many sows did not meet the learning criteria?

**Response: This information has been added.**

L282 “with latency being defined as the time to approach the specific stimulus cue” The authors just said they used an index so which variable was used for statistical analyses is not clear (i.e., raw latencies or JBI?).

**Response: The Judgment Bias Index was created to standardize the different approach speed between sows (i.e., some animals simply always moved slower than others). But as the reviewers point out, this index is not used for further analysis or interpretation, and therefore, we have removed all information about the JBI from this manuscript.**

L305 you mention > 0.6 in the method section.

**Response: The cut-off for ‘strong loading’ will remain 0.6. This sentence has been revised.**

Results

There are several points in the results and presentation of the results that are confusing. First, what is the point of calculating an index if not to use it? I honestly prefer the raw latencies that are much easier to comprehend. If used, the authors need to explore whether responses to positive and negative cues affected response to the ambiguous cue (which seems to have been done later on in the analysis?).

**Response: The Judgment Bias Index was created to standardize the different approach speed between sows (i.e., some animals simply always moved slower than others). But as the reviewers point out, this index is not used for further analysis or interpretation, and therefore, we have removed all information about the JBI from this manuscript.**

Second, in Figure 3A. I do not understand why the authors categorized sows according to their response to the ambiguous cue here. It makes the reader thinking that the analysis is categorical when it is not. Typically, studies using JBT would display this type of graph to show that animals (at the population level) responded to the ambiguous cue as expected (i.e. in average responses are situated between the two learnt stimuli). As is, the figure makes us think that the authors removed the four animals that displayed intermediate responses from the analyses. Please modify this figure either by presenting means for all three locations or individual responses or a combination of both.

**Response: We agree with the reviewer that there is a better way to display this data. The JBI data has been removed from the manuscript, and the original Figure 3A an 3B has also been removed. We have included a new Figure 3, which displays the data points for individual animal approaching the three difference cue locations as well as a mean value for each animal group (optimistic, pessimistic, and undetermined bias).**

“Both positive and negative bias animals approach the positive and negative conditioned stimulus with similar latencies independent of their judgement bias.”

So what’s the point of the index then? In addition, I really don’t like the expressions “positive bias animals” and “negative bias animals” Why not talking about “optimistic” and “pessimistic” animals like the rest of the literature? Alternatively, you could also talk about animals displaying positive and negative expectations.

**Response: We have removed all references and uses of the JBI from the manuscript, and are focusing all analyses and interpretations on the raw latencies. We have also replaced all terms of ‘positive or negative bias” with the terms “optimistic or pessimistic bias” to be consistent with the literature.**

“It appears that these animals failed to exhibit such a preference as the others.”

I am not sure I understand what the authors mean here. A preference for what?

**Response: This sentence has been revised to state, “The response of four sows was intermediate between the more optimistic or more pessimistic responses of others sows, and therefore, were labeled as having an undetermined bias”.**

L365 Can the authors better explain why they did not test the effect of probe location on latencies independently from the other variables?

**Response: Additional probes were not used in the design given the physical limitations of the sows. We have included in the methods that our pregnant sows were not able to be placed in multiple trials, and thus additional probe cues, as they became physically exhausted from standing and walking after 30 minutes. In addition, we argue that there is a significant interaction between probe location and latency of approach can be seen in Figure 3.**

L365 Again, here it feels like the authors are using the raw variables instead of the individual coordinates obtained from the three dimensions of the PCA. What’s the point of the PCA then if not to summarize information? As far as I understand correctly, the model should look like: latency = probe location * aggressiveness + probe location * submissiveness + probe location * activity. If yes, then why do we have 3 variables that were used to assess the trait “aggressiveness” tested separately? Also, it seems that the authors are using different terms for the same variables and it makes it difficult to follow. So maybe I am just getting confused here.

**Response: We have revised this section to clarify that 10 behaviors were compressed using PCA to create 3 composite traits. This way, only one trait score for “aggressiveness” among the sows was compared to approach latencies (instead of 6 separate behavioral comparisons to approach latencies [i.e., bite, chase, displace, etc.]). The behavior of a sow during the ‘litter restraint test’ was removed from the PCA, as it is evaluated as a separate behavioral measure. We have added to this section, “There were no significant effects of the submissive or active/exploratory trait scores on the latency to approach positive, ambiguous or negative stimulus.”**

Figure 4 could also be improved. R2 are missing and usually regressions are represented using squared frame (same width and height) so that regression lines are easier to interpret.

**Response: The graphs of Fig 4 have been revised, and now include r-squared values.**

Looking at the relationship between aggressiveness and JB, I wonder if the authors considered a quadratic model? As displayed it feels like more aggressive sows were more optimistic but less aggressive ones were either optimistic or pessimistic. Thus, not a true linear relationship that is consistent with what Asher found if my memories are correct. This point needs to be discussed.

**Response: Asher et al reported interactions between cue location, personality trait and housing type, but all were described with a simple linear mixed model. We appreciate the reviewer’s point about the variability/noisiness of the latency response as the aggressive/dominant trait score decreases. We believe that given this variability we would need significantly more observations to successfully fit a more complicated model such a quadratic and thus opted to use a simple linear mixed model as others like Asher et al have done before.**

Discussion

“Both positive and negative judgement bias”. It feels odd to phrase it this way (see previous comment). I would suggest that the authors change for individual differences in judgment bias (here and elsewhere in the paper) for two reasons: 1) we don’t know when a positive and a negative bias starts. 2) it puts a lot of emphasis on the extremes and tend to ignore the four sows that expressed true intermediate responses. How should they be considered? as neutral judgment bias? Using “inter-individual differences” will solve these issues and be more consistent with the literature.

**Response: We have also replaced all terms of ‘positive or negative bias’ with the terms “optimistic or pessimistic bias” to be consistent with the literature. We have also included a statement about the ‘undetermined bias’ sows from our study in this section.**

“Perhaps surprisingly, we did not find that animals with more severe physical indicators of poor welfare, such as reduced body condition or increased lesion severity, displayed more negative judgement biases”

Well is this really surprising considering that the authors just explained that lameness and BCS were not really variable in their cohort? These two elements should be removed from all conclusion sentences as the authors don’t know whether more lame or more thin animals would express negative judgment bias. The authors could add a sentence stating that “variation in BCS and Lameness was too small for meaningful analyses” and only comment on the severity of the lesions.

**Response: We have removed the emphasis on body condition and lameness as indicators of welfare condition, and included the suggested statement of the lack variation of these measures.**

L428 is this a continuum though? The authors’ data suggest that submissiveness and aggressiveness are independent (on two axes of the PCA). This is consistent with recent research on dominance in sows, which should be incorporated here.

**Response: The word ‘continuum’ has been removed from this sentence, and previous studies which found that lesion scores among sows is not a direct proxy for aggression measurement have been cited.**

“Taken together, these results suggest that the judgement bias paradigm used in the present study more likely was evaluating a sow’s response to risk as it relates to consistent individual differences in behavior (i.e., personality) rather than exclusively evaluating the subjective mood state (i.e., mental welfare) of the sow” How do you know? Maybe the “personality differences” you assessed reflected or were affected by the overall mood states of the animals.

I’d like the authors to integrate the growing research that explore judgment biases as states and traits. See research being done in dairy calves for more information on this aspect. I think this will help the authors phrase their results.

**Response: We have included more literature which examines judgment bias and personality in other species throughout the discussion. In addition, we have included a short review of the theories behind individual differences in appraisal of stimuli as it relates to state (mood) and trait (affective style) (i.e., Faustino et al., 2015).**

“locomotion-based” well this type of test is typically referred to as a spatial learning task.

**Response: These phrase ‘locomotion based’ has been replace with “spatial learning task”.**

L436 – 458 I agree with the authors. Similar issues were found in laboratory animals when the relationship between stereotypic behaviours and response to jbt were assessed.

**Response: We appreciate the reviewer’s recognition of the relevance for this section.**

L460 “(rats, dogs, parrots)” but you cite studies in pigs, dogs and primates.

**Response: This sentence has been removed.**

L461 Which personality traits though? To my knowledge none of these studies looked at aggressiveness per se. In addition, there is much more in the literature than these three studies and it may help the authors to look more deeply at what has been found in species such as rats, dairy calves, dolphins and many others. Some of these studies specifically looked at dominance, social behaviours, fearfulness etc. All these elements could help the authors better interpreting their results.

**Response: This sentence has been removed.**

I also want to attract the attention of the authors that some work being done in dogs and primates are harder to interpret given that questionnaires are sometimes used in dogs and low sample size are frequent in primates. These methodological differences may render comparisons difficult.

**Response: We agree with the reviewer, that personality determined from human ratings, rather than ethological coding, can be difficult to interpret (or generalize to other animal personality studies). Therefore, we have reduced the reference to such studies and focused on referring to studies with similar methodology as our present study.**

L464 to 473. It is a little difficult to follow the authors here. Please try to rewrite and clarify.

**Response: This paragraph has been rewritten to clarify our point that there are themes within the behavioral syndrome, coping style, and personality literature which link the traits related to survival (i.e. large risk taking for big reward).**

L476 why not, but do the authors have evidence that injuries lead to small differences in subjective state?

**Response: We have removed this section of the sentence to prevent the insinuation that we assume small injuries would lead to differences in mood state.**

L480 yes! Thus, I am sorry to say that calling the behavioural assessment done in these sows “personality traits” is a bit misleading (see previous comments), unless the authors can better justify?

**Response: We have replaced the word ‘personality traits’ with ‘behavioral traits’ throughout the manuscript, but maintain the theme of individual differences in behavior consistent over time were found with the dataset; as we have established this difference using the same study cohort in another peer reviewed study (Horback and Parsons, 2018, *Behavioral Processes*).**

L511 a very recent study in mice support the point being made here (see Jardim et al., 2021)

**Response: The Jardim et al (2021) paper has been incorporated into this sentence.**

L516 yes! You will see that following studies in calves from the same group explored the logic you described at the end of the discussion.

**Response: We have incorporated additional studies on animal personality, judgment bias, and state vs trait of appraisal analysis throughout the introduction and discussion based on the recommendations of the reviewer.**
