# Supplementary material for: Judgement bias of group housed gestating sows predicted by behavioral traits, but not physical measures of welfare
Source: PLoS One. 2022 Feb 25;17(2):e0264258. doi: 10.1371/journal.pone.0264258 (PMC8880926; doi:10.1371/journal.pone.0264258)
Supplement: Attachment — Submitted filename: PLOS ONE revision note #3 CLEAN.docx [file pone.0264258.s003.docx]

Revision note

12/28/2021

PONE-D-21-16018

Judgement bias of group housed gestating sows predicted by personality traits, but not physical measures of welfare

Reviewer #2: First, I want to thank the authors for a great revision of their paper. I think it has greatly improved. I also think the study contributes to highlight a growing problem in the field: what do we do with animal welfare markers that do not correlate? I would like the authors to consider putting more emphasis on this point rather than concluding that JBTs provide limited information. We actually do not know which marker is the most limited. It is also interesting to put forward that when JBT are used in absence of an emotional manipulation, it typically provides information about individual characteristics. I made a few more comments that I’d like the authors to consider (see below).

**Response: We thank the reviewer for providing very helpful feedback to improve this manuscript. We agree that different welfare metrics will have different limitations and is a challenge in elucidating the overall affective state of the animal. In the revised version of this manuscript, we suggest that individual behavioral characteristics could create differential emotional responses that are captured in the JBT. Perhaps an animal’s boldness or risk attributes determine how they perceive a complicated social environment like pen gestation and as such influences their emotional response. We have amended the introduction and discussion to place more emphasis on the role of cognitive bias testing; that is, how do we interpret response latency given the possibility that different factors (eg behavioral traits and lesion) may be differentially contributing to the animal’s affective state.**

Abstract:

The authors make a very strong claim here (i.e., that JBT may not “be the most appropriate method for evaluating affective state”), but I am not convinced. This argument is based on 1) high variability in responses to JBT in enriched animals, 2) a positive relationship with aggressiveness and 3) a lack of relationship with other so-called welfare markers (e.g., mostly the number of lesions after regrouping).

First, yes, there is high variability, but this does not mean swine kept in barren conditions would not score worse. Second, yes it correlates with aggressiveness, which makes sense given that aggressiveness often correlates with proactive coping styles, impulsivity and optimism. I’m not sure if the authors saw this recent review (Lecorps et al., 2021. Negative expectations and vulnerability to stressors in animals) but it seems very relevant for this study! Third, are these physical indicators truly reflecting what animals feel or their welfare state? Are only unhealthy humans feeling terrible?

**Response: Not sure, what the reviewer means in terms of sows in barren conditions scoring worse. Our goal is not the response of the group but that fact that several individual animals essentially interpret the ambiguous stimulus as the negative cue. I do not know how these animals could score worse even if housed in barren conditions. However, this does speaks towards the notion that personality attributes could be creating the emotional experience captured in this JBT. We also agree that physical compromise could, but necessarily, result in negative affective states and tried to better explain the observed disconnect between lesions scores and JBT outcomes. The abstract has been modified to capture these nuances.**

I think the authors should discuss more this aspect because it is a very important one and they are not the only ones that failed to show correlations between psychological and physical markers of well-being (e.g., Lecorps et al. 2021. Regrouping induces anhedonia-like responses in dairy heifers; in that study no correlations between anhedonia-like behaviours and the number of agonistic interactions received during regrouping were found). Importantly, I think this does not necessarily mean that the methodologies do not work well but rather that our conception of welfare is somewhat challenged. I would like the authors to include this aspect (i.e., the difficulty to correlate different welfare parameters) in their conclusions. As is, the claims are not largely supported by the authors’ dataset.

**Response: We appreciate the reviewer’s request to make this point more explicit in our paper. We have added the following lines to our discussion to highlight that this disconnect between physical and psychological welfare assessments (lines 441-449): “Current welfare assessments for breeding sows focus on physical ailments (e.g., lameness, body condition and skin lesions), as well as being the target of aggressive behaviors (e.g., bites, scratches, displacement). However, given that recent work in dairy heifer welfare has reported a lack of correlation between behavioral indicators of anhedonia and the number of agonistic interactions received during regrouping (Lecorps et al., 2021), there is a need to carefully critique our assumptions of how welfare is experienced by the animal. If we are using judgement bias testing as an indicator of subjective mental welfare of animals, we also need to better understand how, and if, the degree and duration of physical comprise contribute to an animal’s psychological welfare state.”**

L54 There are several reviews addressing cumulative affective experiences and mood-related disorders in farm animals (Poirier et al., 2019, Bateson and Poirier, 2019, Lecorps et al., 2021, McLellan and Mason, 2021), maybe worth mentioning at least one of these so that the interested reader can learn more?

**Response: We have revised this sentence to clarify that little attention is currently being given to breeding sow affective state (and not farm animals in general), but we have added a citation to this sentence to allow the reader to know of the growth of knowledge on the subject of affective states in farm animals.**

L69 to 73 I haven’t read this particular contribution of Stamps but I find some of the arguments quite surprising. For instance, what do “early in life” or “biologically based” mean? Are all traits more clearly expressed in “novel and unpredictable situations” or just the ones we care to measure in animals? It makes perfect sense if we assess neophobia, boldness or pessimism but does it make sense for sociability and aggressiveness? In my opinion, these are risky claims, but the authors are of course free to keep this section if they feel it helps with the narrative.

**Response: This summary of personality has been revised to reduce any claims of genetically-linked traits, or strict consistency over an organism’s lifetime. This sentence now reads, “Personality can be described as characteristics of behavioral reactions such as intensity, speed, response threshold, latency and recovery time, which are most clearly expressed in novel and unpredictable situations, and are relatively stable throughout an individual’s development.”**

L97 “Given that there is limited information on the use and validity of judgement bias testing to evaluate affective states in sexually-mature sows, there is a need to investigate possible influences on performance in this cognitive task. As greater emphasis is being placed on the welfare of commercial sows, because they live the longest out of all production swine and thus have the greatest capacity to incur chronic suffering if their welfare is compromised, investigating new tools for welfare assessment is crucial.”

The second sentence could be deleted as it basically repeats what is said earlier (L85). I’m still not sure to understand the aims as currently formulated.

**Response: The second sentence has been removed to prevent redundancy. A sentence clarifying the aim of this study has been added in its place.**

L102 to 108 maybe this part belongs to the method section?

**Response: These sentences have been removed as they summarize the methods of this study.**

L112 “if judgement bias reflects the affective state of an animal (i.e., biological health and mental state), then it follows that physical measurements of welfare (i.e., skin lesions, body condition) should also impact performance in judgement bias task”

As argued before this may not be necessarily true. However this is a good working hypothesis.

**Response: We have kept this portion of the hypothesis in place, as we agree with the reviewer that physical health may not be the best predictor of subjective mental state; this notion is assumed in some animal emotion/welfare literature.**

L278 For clarity sake I would start this sentence by “Additional probe cues”.

**Response: This change has been made.**

L358 to 363 this basically repeats the previous sentences, delete?

**Response: These sentences have been deleted.**

Figure 3 seems to have changed based on caption changes but I could not access it (neither any other figures).

**Response: We do not know why the reviewer could not access the new Figure 3 (offered below)**

Figure 4 has also been changed according to the authors’ response, but I could not see the changes.

**Response: We do not know why the reviewer could not access the new Figure 4 (offered below)**

L446 affective or coping style?

**Response: This change has been made.**

L454 “we need to reconcile inconsistencies when comparing the physical and assumed psychological welfare state.” I’d push the authors to consider that these may not be inconsistencies. Health being a poor predictor of psychological well-being in humans, it may well be the same in animals. However, I agree with the authors that lameness should, in theory, correlate with judgment bias given its severe impact on the animals’ quality of life.

**Response: We agree with the reviewer on this suggestion, and have added a sentence the following sentence, “If we are using judgement bias testing as an indicator of subjective mental welfare of animals, we also need to better understand how, and if, the degree and duration of physical comprise contribute to an animal’s psychological welfare state.”**

L479 there is a problem with this sentence (missing word?).

**Response: This sentence has been removed.**

L519 “it can be argued”. There are several typos along the manuscript that the authors will want to address before publication.

**Response: The word “be” has been added.**
