# Supplementary material for: Judgement bias of group housed gestating sows predicted by behavioral traits, but not physical measures of welfare
Source: PLoS One. 2022 Feb 25;17(2):e0264258. doi: 10.1371/journal.pone.0264258 (PMC8880926; doi:10.1371/journal.pone.0264258)
Supplement: Peer review history

# Peer review: Decision Letter 0

**DOI:** 10.1371/journal.pone.0264258.r001

**Authors:** Miquel Llorente

22 Sep 2021

PONE-D-21-16018Judgement bias of group housed gestating sows predicted by personality traits, but not physical measures of welfarePLOS ONE

Dear Dr. Horback,

Dear Dr. Kristina Horback,

First, sorry for the delay in our decision. I recognise that the impact of COVID-19 may affect the ability of our reviewers to return your revised manuscript. I have now received the report of the both reviewers.

In the light of the reviewers' comments (R1 and R2) and my own reflection, and after careful consideration, we have decided that your manuscript does not meet our criteria for publication in its current form and requires major revision. After careful consideration, we feel that it has merit but does not fully meet PLOS ONE’s publication criteria as it currently stands. Therefore, we invite you to submit a revised version of the manuscript that addresses the points raised during the review process.

We look forward to receiving your revised manuscript

Yours sincerely,

Please submit your revised manuscript by Oct 30 2021 11:59PM. If you will need more time than this to complete your revisions, please reply to this message or contact the journal office at plosone@plos.org. When you're ready to submit your revision, log on to https://www.editorialmanager.com/pone/ and select the 'Submissions Needing Revision' folder to locate your manuscript file.

Please include the following items when submitting your revised manuscript:

- A rebuttal letter that responds to each point raised by the academic editor and reviewer(s). You should upload this letter as a separate file labeled 'Response to Reviewers'.
- A marked-up copy of your manuscript that highlights changes made to the original version. You should upload this as a separate file labeled 'Revised Manuscript with Track Changes'.
- An unmarked version of your revised paper without tracked changes. You should upload this as a separate file labeled 'Manuscript'.

If you would like to make changes to your financial disclosure, please include your updated statement in your cover letter. Guidelines for resubmitting your figure files are available below the reviewer comments at the end of this letter.

If applicable, we recommend that you deposit your laboratory protocols in protocols.io to enhance the reproducibility of your results. Protocols.io assigns your protocol its own identifier (DOI) so that it can be cited independently in the future. For instructions see: https://journals.plos.org/plosone/s/submission-guidelines#loc-laboratory-protocols. Additionally, PLOS ONE offers an option for publishing peer-reviewed Lab Protocol articles, which describe protocols hosted on protocols.io. Read more information on sharing protocols at https://plos.org/protocols?utm_medium=editorial-email&utm_source=authorletters&utm_campaign=protocols.

We look forward to receiving your revised manuscript.

Kind regards,

Miquel Llorente, PhD

Academic Editor

PLOS ONE

Journal Requirements

When submitting your revision, we need you to address these additional requirements.

1. Please ensure that your manuscript meets PLOS ONE's style requirements, including those for file naming. The PLOS ONE style templates can be found at

https://journals.plos.org/plosone/s/file?id=wjVg/PLOSOne_formatting_sample_main_body.pdf and https://journals.plos.org/plosone/s/file?id=ba62/PLOSOne_formatting_sample_title_authors_affiliations.pdf

2, We note that the grant information you provided in the ‘Funding Information’ and ‘Financial Disclosure’ sections do not match.

When you resubmit, please ensure that you provide the correct grant numbers for the awards you received for your study in the ‘Funding Information’ section.

3. Thank you for stating the following in the Acknowledgments Section of your manuscript:

“The authors would like to thank the summer research interns of the Swine Research and Teaching Center for their assistance in data collection. This work was supported in part by Kraft Foods Oscar Mayer, Pig Improvement Company, Pennsylvania Pork Producers Council, Pennsylvania Soybean Board, Pennsylvania Department of Agriculture, and the American Society for the Prevention of Cruelty to Animals”

We note that you have provided additional information within the Acknowledgements Section that is not currently declared in your Funding Statement. Please note that funding information should not appear in the Acknowledgments section or other areas of your manuscript. We will only publish funding information present in the Funding Statement section of the online submission form.

Please remove any funding-related text from the manuscript and let us know how you would like to update your Funding Statement. Currently, your Funding Statement reads as follows:

“This work was supported in part by Kraft Foods Oscar Mayer, Pig Improvement Company, Pennsylvania Pork Producers Council, Pennsylvania Soybean Board, Pennsylvania Department of Agriculture, and the American Society for the Prevention of Cruelty to Animals. The funders had no role in study design, data collection and analysis, decision to publish, or preparation of the manuscript”

Please include your amended statements within your cover letter; we will change the online submission form on your behalf.

[Note: HTML markup is below. Please do not edit.]

Reviewers' comments:

Reviewer's Responses to Questions

**Comments to the Author**

1. Is the manuscript technically sound, and do the data support the conclusions?

The manuscript must describe a technically sound piece of scientific research with data that supports the conclusions. Experiments must have been conducted rigorously, with appropriate controls, replication, and sample sizes. The conclusions must be drawn appropriately based on the data presented. 

Reviewer #1: Yes

Reviewer #2: Partly

**********

2. Has the statistical analysis been performed appropriately and rigorously? 

Reviewer #1: Yes

Reviewer #2: Yes

**********

3. Have the authors made all data underlying the findings in their manuscript fully available?

The PLOS Data policy requires authors to make all data underlying the findings described in their manuscript fully available without restriction, with rare exception (please refer to the Data Availability Statement in the manuscript PDF file). The data should be provided as part of the manuscript or its supporting information, or deposited to a public repository. For example, in addition to summary statistics, the data points behind means, medians and variance measures should be available. If there are restrictions on publicly sharing data—e.g. participant privacy or use of data from a third party—those must be specified.

Reviewer #1: Yes

Reviewer #2: Yes

**********

4. Is the manuscript presented in an intelligible fashion and written in standard English?

PLOS ONE does not copyedit accepted manuscripts, so the language in submitted articles must be clear, correct, and unambiguous. Any typographical or grammatical errors should be corrected at revision, so please note any specific errors here.

Reviewer #1: Yes

Reviewer #2: Yes

**********

5. Review Comments to the Author

Please use the space provided to explain your answers to the questions above. You may also include additional comments for the author, including concerns about dual publication, research ethics, or publication ethics. (Please upload your review as an attachment if it exceeds 20,000 characters)

Reviewer #1: This is an important subject, to challenge the recent tendency to over-interpret results from cognitive bias paradigms as de facto evidence of good or bad welfare. It would be nice to see consideration of alternative explanations for cognitive bias results become the norm.

Reviewer #2: This paper aims at exploring what factors influence performance in judgement bias testing in group-housed sows with an emphasis on personality traits and measures of physical aggressions.

This is an important area of research given the need for a better understanding of affective states of farmed animals and how it can successfully relate to other welfare measures.

I think the study has merits, but I feel it could really be improved. I spent quite some time making both general and detailed comments that I hope will help the authors.

General comments

• The literature considered in this manuscript is rather incomplete as work on other species (dairy calves and rats) has not been integrated. I would strongly recommend including what has been done in other species given that only a few studies have explored individual variation in response to judgment bias. For now, the authors do not really refer to studies exploring this link but rather mention their existence. I think the integration of these studies will provide more context and should help the authors better communicate their results, especially in terms of how moods can be considered both as reflecting emotional states and/or personality differences.

• The rationale behind the research question could be improved. For now, the reader is left with limited information on why the authors did this study and why it is important.

• I’d like the authors to discuss more some limitations of their study. For instance, the authors used only one ambiguous cue presented only once, which is at odds with the literature. The authors need to explain why they made that choice and more effectively explain how this could have affected their results (i.e., animals may be more incline to react to ambiguous cues as novel element in their environment on the first exposure vs. on repeated exposures).

• Many scientists would argue that personality traits need to be assessed over time and contexts. In addition, tests were not all standardized (e.g., the social interaction test was more a regrouping experiment), which is a limitation of the study. I would recommend to keep the concept of personality for the discussion but I may be convinced otherwise if the authors clearly stress the consistency of their outcome variables.

• What is the point of the JBI? I would remove this part as the authors do not seem to use it for further analyses.

Abstract

L21 states?

L22 I feel the authors should give more information to the reader so that it is easier to grasp how JBTs work. I would add elements explaining pessimistic and optimistic responses.

L26 Maybe better to split this sentence?

L36 I would tone down the conclusion as the authors did not manipulate affective states in this study.

Introduction

“Due to both government legislation and consumer demands, the practice of housing of gestating gilts and sows as individuals in stalls has been banned in many developed countries, including the EU1, Australia, New Zealand, Canada, South Africa and ten states in the United States2,3”

This sentence could be read as stalls are forbidden in these countries. In practice animals can still be confined during the lactation period and sometimes for a few weeks during the gestation period. Can the authors add clarification here?

“The personality of an organism refers to the relatively consistent behavioral reactions of an organism across contexts and is most clearly expressed in novel and unpredictable situations17”

This sentence is not super clear + avoid repetitions (maybe replace organism by individuals or animals). Overall the authors do not clearly articulate how their measures can be used to reflect personality traits in sows.

“and the expression of pain21” Again there is more recent work that would better fit your article as variation in jbt responses has been linked to pain in calves (see Lecorps et al., 2020).

L70 to 74: I would push the authors to also consider the work being done in rats and dairy calves.

L75 the logic needs some work in this paragraph. The authors start by saying that there is not enough research being done on affective states (from this paragraph it feels like the authors say that research on affective states in pigs are limited to judgment bias studies). Then they say that there is not enough research being done on personality traits. However, they do not really stress out why we need to explore the relationship between response to jbt (hypothesized to reflect on mood) and personality traits. Why is this important?

One or two additional sentences would help the reader following the point made by the authors.

Overall, I think this is one of the points I’d like the authors to focus on. The conceptualization of the study and rationale for the question asked is not very clear in the introduction. The paper would be much stronger if these points were improved.

L95 will impact response to judgment bias tests

Methods

L158 a dot is missing

Personality tests used seem different from the rest of the literature. Can the authors comment on that? What is typically done? Why not mimicking what Asher et al 2016 (one of the key reference for this work) did?

I like the fact that the procedures intended to use routine procedures happening to piglets/sows in commercial settings, but I think this choice needs to be justified and explained.

L171 An illustration of my doubts regarding the first paragraph of the introduction. The readers may be led to think that the ban of stalls/crates means animals are no longer kept in confinement during their life, which is not the case. However, this still happens at various times.

L205 “sow moves easily with little inducement. She is comfortable on all her feet” I would link these two sentences, otherwise it is a little confusing. Same for the following sentences. Another option would be to have a table for this.

“Sows were considered trained if they approached the positive stimulus within 20 s for 100% of the positive trials and if they did not approach the negative stimulus at least 50 s for at least 80% of the negative trials.”

Can the authors explain why they chose these criteria? Do they come from previous studies?

L254 Why only one ambiguous trial? This is very unusual as most JBT studies would use at least multiple exposure to the same ambiguous cue or to multiple cues. How confident are the authors that this was enough? I feel this needs to be justified.

L259 It is not clear if the authors mean they used all variables collected in all the different tests (except JBT) or just “during pen introduction” (i.e. social interactions).

L268 please state here how many sows did not meet the learning criteria?

L282 “with latency being defined as the time to approach the specific stimulus cue” The authors just said they used an index so which variable was used for statistical analyses is not clear (i.e., raw latencies or JBI?).

L305 you mention > 0.6 in the method section.

Results

There are several points in the results and presentation of the results that are confusing.

First, what is the point of calculating an index if not to use it? I honestly prefer the raw latencies that are much easier to comprehend. If used, the authors need to explore whether responses to positive and negative cues affected response to the ambiguous cue (which seems to have been done later on in the analysis?).

Second, in Figure 3A. I do not understand why the authors categorized sows according to their response to the ambiguous cue here. It makes the reader thinking that the analysis is categorical when it is not. Typically, studies using JBT would display this type of graph to show that animals (at the population level) responded to the ambiguous cue as expected (i.e. in average responses are situated between the two learnt stimuli). As is, the figure makes us think that the authors removed the four animals that displayed intermediate responses from the analyses. Please modify this figure either by presenting means for all three locations or individual responses or a combination of both.

“Both positive and negative bias animals approach the positive and negative conditioned stimulus with similar latencies independent of their judgement bias.”

So what’s the point of the index then? In addition, I really don’t like the expressions “positive bias animals” and “negative bias animals” Why not talking about “optimistic” and “pessimistic” animals like the rest of the literature? Alternatively, you could also talk about animals displaying positive and negative expectations.

“It appears that these animals failed to exhibit such a preference as the others.”

I am not sure I understand what the authors mean here. A preference for what?

L365 Can the authors better explain why they did not test the effect of probe location on latencies independently from the other variables?

L365 Again, here it feels like the authors are using the raw variables instead of the individual coordinates obtained from the three dimensions of the PCA. What’s the point of the PCA then if not to summarize information?

As far as I understand correctly, the model should look like: latency = probe location * aggressiveness + probe location * submissiveness + probe location * activity.

If yes, then why do we have 3 variables that were used to assess the trait “aggressiveness” tested separately?

Also, it seems that the authors are using different terms for the same variables and it makes it difficult to follow. So maybe I am just getting confused here.

Figure 4 could also be improved. R2 are missing and usually regressions are represented using squared frame (same width and height) so that regression lines are easier to interpret.

Looking at the relationship between aggressiveness and JB, I wonder if the authors considered a quadratic model? As displayed it feels like more aggressive sows were more optimistic but less aggressive ones were either optimistic or pessimistic. Thus, not a true linear relationship that is consistent with what Asher found if my memories are correct. This point needs to be discussed.

Discussion

“Both positive and negative judgement bias”. It feels odd to phrase it this way (see previous comment). I would suggest that the authors change for individual differences in judgment bias (here and elsewhere in the paper) for two reasons: 1) we don’t know when a positive and a negative bias starts. 2) it puts a lot of emphasis on the extremes and tend to ignore the four sows that expressed true intermediate responses. How should they be considered? as neutral judgment bias? Using “inter-individual differences” will solve these issues and be more consistent with the literature.

“Perhaps surprisingly, we did not find that animals with more severe physical indicators of poor welfare, such as reduced body condition or increased lesion severity, displayed more negative judgement biases”

Well is this really surpsising considering that the authors just explained that lameness and BCS were not really variable in their cohort?

These two elements should be removed from all conclusion sentences as the authors don’t know whether more lame or more thin animals would express negative judgment bias. The authors could add a sentence stating that “variation in BCS and Lameness was too small for meaningful analyses” and only comment on the severity of the lesions.

L428 is this a continuum though? The authors’ data suggest that submissiveness and aggressiveness are independent (on two axes of the PCA). This is consistent with recent research on dominance in sows, which should be incorporated here.

“Taken together, these results suggest that the judgement bias paradigm used in the present study more likely was evaluating a sow’s response to risk as it relates to consistent individual differences in behavior (i.e., personality) rather than exclusively evaluating the subjective mood state (i.e., mental welfare) of the sow”

How do you know? Maybe the “personality differences” you assessed reflected or were affected by the overall mood states of the animals.

I’d like the authors to integrate the growing research that explore judgment biases as states and traits. See research being done in dairy calves for more information on this aspect. I think this will help the authors phrase their results.

“locomotion-based” well this type of test is typically referred to as a spatial learning task.

L436 – 458 I agree with the authors. Similar issues were found in laboratory animals when the relationship between stereotypic behaviours and response to jbt were assessed.

L460 “(rats, dogs, parrots)” but you cite studies in pigs, dogs and primates.

L461 Which personality traits though? To my knowledge none of these studies looked at aggressiveness per se.

In addition, there is much more in the literature than these three studies and it may help the authors to look more deeply at what has been found in species such as rats, dairy calves, dolphins and many others. Some of these studies specifically looked at dominance, social behaviours, fearfulness etc. All these elements could help the authors better interpreting their results.

I also want to attract the attention of the authors that some work being done in dogs and primates are harder to interpret given that questionnaires are sometimes used in dogs and low sample size are frequent in primates. These methodological differences may render comparisons difficult.

L464 to 473. It is a little difficult to follow the authors here. Please try to rewrite and clarify.

L476 why not, but do the authors have evidence that injuries lead to small differences in subjective state?

L480 yes! Thus, I am sorry to say that calling the behavioural assessment done in these sows “personality traits” is a bit misleading (see previous comments), unless the authors can better justify?

L511 a very recent study in mice support the point being made here (see Jardim et al., 2021)

L516 yes! You will see that following studies in calves from the same group explored the logic you described at the end of the discussion.

**********

6. PLOS authors have the option to publish the peer review history of their article (what does this mean?). If published, this will include your full peer review and any attached files.

If you choose “no”, your identity will remain anonymous but your review may still be made public.

**Do you want your identity to be public for this peer review?** For information about this choice, including consent withdrawal, please see our Privacy Policy.

Reviewer #1: No

Reviewer #2: No

[NOTE: If reviewer comments were submitted as an attachment file, they will be attached to this email and accessible via the submission site. Please log into your account, locate the manuscript record, and check for the action link "View Attachments". If this link does not appear, there are no attachment files.]

While revising your submission, please upload your figure files to the Preflight Analysis and Conversion Engine (PACE) digital diagnostic tool, https://pacev2.apexcovantage.com/. PACE helps ensure that figures meet PLOS requirements. To use PACE, you must first register as a user. Registration is free. Then, login and navigate to the UPLOAD tab, where you will find detailed instructions on how to use the tool. If you encounter any issues or have any questions when using PACE, please email PLOS at figures@plos.org. Please note that Supporting Information files do not need this step.

# Author comment: Author response to Decision Letter 0

**DOI:** 10.1371/journal.pone.0264258.r002

29 Oct 2021

Revision note

PONE-D-21-16018

Judgement bias of group housed gestating sows predicted by personality traits, but not physical measures of welfare

Editor:

When submitting your revision, we need you to address these additional requirements.

1. Please ensure that your manuscript meets PLOS ONE's style requirements, including those for file naming. 

Response: PLOS ONE style requirements were addressed.

2, We note that the grant information you provided in the ‘Funding Information’ and ‘Financial Disclosure’ sections do not match. When you resubmit, please ensure that you provide the correct grant numbers for the awards you received for your study in the ‘Funding Information’ section.

Response: This has been addressed during re-submission.

3. Thank you for stating the following in the Acknowledgments Section of your manuscript:

“The authors would like to thank the summer research interns of the Swine Research and Teaching Center for their assistance in data collection. This work was supported in part by Kraft Foods Oscar Mayer, Pig Improvement Company, Pennsylvania Pork Producers Council, Pennsylvania Soybean Board, Pennsylvania Department of Agriculture, and the American Society for the Prevention of Cruelty to Animals”

We note that you have provided additional information within the Acknowledgements Section that is not currently declared in your Funding Statement. Please note that funding information should not appear in the Acknowledgments section or other areas of your manuscript. We will only publish funding information present in the Funding Statement section of the online submission form. Please remove any funding-related text from the manuscript and let us know how you would like to update your Funding Statement. Currently, your Funding Statement reads as follows:

“This work was supported in part by Kraft Foods Oscar Mayer, Pig Improvement Company, Pennsylvania Pork Producers Council, Pennsylvania Soybean Board, Pennsylvania Department of Agriculture, and the American Society for the Prevention of Cruelty to Animals. The funders had no role in study design, data collection and analysis, decision to publish, or preparation of the manuscript”

Please include your amended statements within your cover letter; we will change the online submission form on your behalf.

Response: We have removed all references to funding support from the acknowledgments and included this information in the funding statement.

Reviewer #1: This is an important subject, to challenge the recent tendency to over-interpret results from cognitive bias paradigms as de facto evidence of good or bad welfare. It would be nice to see consideration of alternative explanations for cognitive bias results become the norm.

Reviewer #2: This paper aims at exploring what factors influence performance in judgement bias testing in group-housed sows with an emphasis on personality traits and measures of physical aggressions. This is an important area of research given the need for a better understanding of affective states of farmed animals and how it can successfully relate to other welfare measures. I think the study has merits, but I feel it could really be improved. I spent quite some time making both general and detailed comments that I hope will help the authors.

General comments

• The literature considered in this manuscript is rather incomplete as work on other species (dairy calves and rats) has not been integrated. I would strongly recommend including what has been done in other species given that only a few studies have explored individual variation in response to judgment bias. For now, the authors do not really refer to studies exploring this link but rather mention their existence. I think the integration of these studies will provide more context and should help the authors better communicate their results, especially in terms of how moods can be considered both as reflecting emotional states and/or personality differences.

Response: Previous studies which examine personality, coping style, or behavioral syndrome in a variety of specie, as it relates to risk-taking, appraisal or judgment bias, have been incorporated throughout the introduction and discussion.

• The rationale behind the research question could be improved. For now, the reader is left with limited information on why the authors did this study and why it is important.

Response: The last sentence in the second to last paragraph has been revised to clarify why this study was conducted with this particular population of swine.

• I’d like the authors to discuss more some limitations of their study. For instance, the authors used only one ambiguous cue presented only once, which is at odds with the literature. The authors need to explain why they made that choice and more effectively explain how this could have affected their results (i.e., animals may be more incline to react to ambiguous cues as novel element in their environment on the first exposure vs. on repeated exposures).

Response: The reasons for only one ambiguous cue (animal fatigue, chronic animal hunger) have been incorporated into the methods. We have addressed the limitation of one ambiguous trial in the discussion with greater details; including ways to improve upon our study design in the future.

• Many scientists would argue that personality traits need to be assessed over time and contexts. In addition, tests were not all standardized (e.g., the social interaction test was more a regrouping experiment), which is a limitation of the study. I would recommend to keep the concept of personality for the discussion but I may be convinced otherwise if the authors clearly stress the consistency of their outcome variables.

Response: We agree with this critique. We have replace the word ‘personality traits’ with ‘behavioral traits’ throughout the manuscript, but maintain the theme of individual differences in behavior consistent over time were found with the dataset; as we have established this difference using the same study cohort in another peer reviewed study (Horback and Parsons, 2018, Behavioral Processes).

• What is the point of the JBI? I would remove this part as the authors do not seem to use it for further analyses.

Response: The Judgment Bias Index was created to control for differences in the approach speed between sows (i.e., some animals simply always moved slower than others). However, in this study there was not that much difference in locomotion rates between sows and hence why as the reviewers point out, this index is not used for further analysis or interpretation. As such we have removed all information about the JBI from this manuscript.

Abstract

L21 states?

Response: The ‘s’ has been added.

L22 I feel the authors should give more information to the reader so that it is easier to grasp how JBTs work. I would add elements explaining pessimistic and optimistic responses.

Response: Sentences have been added to explain the optimistic/pessimistic interpretation of animal behavior in JBTs.

L26 Maybe better to split this sentence?

Response: This sentence has been split into two.

L36 I would tone down the conclusion as the authors did not manipulate affective states in this study.

Response: This final sentence to the abstract has been revised to reduce the strengths of conclusions to be taken from our data.

Introduction

“Due to both government legislation and consumer demands, the practice of housing of gestating gilts and sows as individuals in stalls has been banned in many developed countries, including the EU1, Australia, New Zealand, Canada, South Africa and ten states in the United States2,3”

This sentence could be read as stalls are forbidden in these countries. In practice animals can still be confined during the lactation period and sometimes for a few weeks during the gestation period. Can the authors add clarification here?

Response: This sentence has been revised.

“The personality of an organism refers to the relatively consistent behavioral reactions of an organism across contexts and is most clearly expressed in novel and unpredictable situations17”

This sentence is not super clear + avoid repetitions (maybe replace organism by individuals or animals). Overall the authors do not clearly articulate how their measures can be used to reflect personality traits in sows.

Response: We have included a more thorough definition of personality and included a segue sentence to describe how tests to evaluate individual differences in behavior should be novel or unpredictable to reveal high-arousal responses.

“and the expression of pain21” Again there is more recent work that would better fit your article as variation in jbt responses has been linked to pain in calves (see Lecorps et al., 2020).

Response: This source has been incorporated to the introduction. 

L70 to 74: I would push the authors to also consider the work being done in rats and dairy calves.

Response: Given that there is work done relating personality to performance in JBTs for many other species (dolphins, mice, rats, dairy cows, etc.), we have decided to expand on this relationship in the discussion, rather than elongate the introduction. This sentence does state that the following studies are examples of this relationship.

L75 the logic needs some work in this paragraph. The authors start by saying that there is not enough research being done on affective states (from this paragraph it feels like the authors say that research on affective states in pigs are limited to judgment bias studies). Then they say that there is not enough research being done on personality traits. However, they do not really stress out why we need to explore the relationship between response to jbt (hypothesized to reflect on mood) and personality traits. Why is this important? One or two additional sentences would help the reader following the point made by the authors. Overall, I think this is one of the points I’d like the authors to focus on. The conceptualization of the study and rationale for the question asked is not very clear in the introduction. The paper would be much stronger if these points were improved.

Response: This paragraph has been revised to include research on assessing swine affective states other than JBTs, as well as to highlight the lack of information on breeding sow personality (instead of piglet personality). Sentences were added to clarify the need for more information on if/how sow personality influences performance in JBT (as little is known about this breeding population).

L95 will impact response to judgment bias tests

Response: This change has been made.

Methods

L158 a dot is missing

Personality tests used seem different from the rest of the literature. Can the authors comment on that? What is typically done? Why not mimicking what Asher et al 2016 (one of the key reference for this work) did? I like the fact that the procedures intended to use routine procedures happening to piglets/sows in commercial settings, but I think this choice needs to be justified and explained.

Response: A sentence has been added to explicitly state and defend the use of these tests which can be incorporated into routine animal husbandry, for a more generalizable and practical use. It should also be noted that the study population is different between our study and Asher et al. In this manuscript, we follow animals from 5 weeks of age to just beyond conception of their second litter (a 50 week or more time span) whereas Asher et al focused on grower pigs that varied in age over a 6 week period from 4 to 10 weeks of age. The personality test that are used here are previously described in the following publications (Horback and Parsons, 2016 & 2018).

L171 An illustration of my doubts regarding the first paragraph of the introduction. The readers may be led to think that the ban of stalls/crates means animals are no longer kept in confinement during their life, which is not the case. However, this still happens at various times.

Response: The first paragraph of the introduction has been modified to reduce the insinuation that sows are never in stalls or crates.

L205 “sow moves easily with little inducement. She is comfortable on all her feet” I would link these two sentences, otherwise it is a little confusing. Same for the following sentences. Another option would be to have a table for this.

Response: The description of the lameness scale was revised to have one sentence per score to reduce confusion.

“Sows were considered trained if they approached the positive stimulus within 20 s for 100% of the positive trials and if they did not approach the negative stimulus at least 50 s for at least 80% of the negative trials.”

Can the authors explain why they chose these criteria? Do they come from previous studies?

Response: These criteria are based on average walking speed of the sows and a clear break in the performance of the sows looking at the data (explained in Horback & Parsons, 2018, Behavioral Processes). This explanation has been added.

L254 Why only one ambiguous trial? This is very unusual as most JBT studies would use at least multiple exposure to the same ambiguous cue or to multiple cues. How confident are the authors that this was enough? I feel this needs to be justified.

Response: We understand this concern as all other spatial judgement bias tasks include probe cues in the assessment. We have included in the methods that our pregnant sows were not able to be placed in multiple trials, and thus additional probe cues, as they became physically exhausted from standing and walking after 30 minutes. Not described in the manuscript, but for the reviewers’ knowledge, we have developed a non-strenuous activity (joystick movement from stationary/sitting animal) to allow for probe cues to be used in future judgment bias tasks with breeding sows. 

L259 It is not clear if the authors mean they used all variables collected in all the different tests (except JBT) or just “during pen introduction” (i.e. social interactions).

Response: This sentence has ben revised to clarify that it is only the behaviors during introduction to pen as adults that were placed in PCA.

L268 please state here how many sows did not meet the learning criteria?

Response: This information has been added.

L282 “with latency being defined as the time to approach the specific stimulus cue” The authors just said they used an index so which variable was used for statistical analyses is not clear (i.e., raw latencies or JBI?).

Response: The Judgment Bias Index was created to standardize the different approach speed between sows (i.e., some animals simply always moved slower than others). But as the reviewers point out, this index is not used for further analysis or interpretation, and therefore, we have removed all information about the JBI from this manuscript.

L305 you mention > 0.6 in the method section.

Response: The cut-off for ‘strong loading’ will remain 0.6. This sentence has been revised.

Results

There are several points in the results and presentation of the results that are confusing. First, what is the point of calculating an index if not to use it? I honestly prefer the raw latencies that are much easier to comprehend. If used, the authors need to explore whether responses to positive and negative cues affected response to the ambiguous cue (which seems to have been done later on in the analysis?).

Response: The Judgment Bias Index was created to standardize the different approach speed between sows (i.e., some animals simply always moved slower than others). But as the reviewers point out, this index is not used for further analysis or interpretation, and therefore, we have removed all information about the JBI from this manuscript.

Second, in Figure 3A. I do not understand why the authors categorized sows according to their response to the ambiguous cue here. It makes the reader thinking that the analysis is categorical when it is not. Typically, studies using JBT would display this type of graph to show that animals (at the population level) responded to the ambiguous cue as expected (i.e. in average responses are situated between the two learnt stimuli). As is, the figure makes us think that the authors removed the four animals that displayed intermediate responses from the analyses. Please modify this figure either by presenting means for all three locations or individual responses or a combination of both.

Response: We agree with the reviewer that there is a better way to display this data. The JBI data has been removed from the manuscript, and the original Figure 3A an 3B has also been removed. We have included a new Figure 3, which displays the data points for individual animal approaching the three difference cue locations as well as a mean value for each animal group (optimistic, pessimistic, and undetermined bias). 

“Both positive and negative bias animals approach the positive and negative conditioned stimulus with similar latencies independent of their judgement bias.”

So what’s the point of the index then? In addition, I really don’t like the expressions “positive bias animals” and “negative bias animals” Why not talking about “optimistic” and “pessimistic” animals like the rest of the literature? Alternatively, you could also talk about animals displaying positive and negative expectations.

Response: We have removed all references and uses of the JBI from the manuscript, and are focusing all analyses and interpretations on the raw latencies. We have also replaced all terms of ‘positive or negative bias” with the terms “optimistic or pessimistic bias” to be consistent with the literature.

“It appears that these animals failed to exhibit such a preference as the others.”

I am not sure I understand what the authors mean here. A preference for what?

Response: This sentence has been revised to state, “The response of four sows was intermediate between the more optimistic or more pessimistic responses of others sows, and therefore, were labeled as having an undetermined bias”.

L365 Can the authors better explain why they did not test the effect of probe location on latencies independently from the other variables?

Response: Additional probes were not used in the design given the physical limitations of the sows. We have included in the methods that our pregnant sows were not able to be placed in multiple trials, and thus additional probe cues, as they became physically exhausted from standing and walking after 30 minutes. In addition, we argue that there is a significant interaction between probe location and latency of approach can be seen in Figure 3. 

L365 Again, here it feels like the authors are using the raw variables instead of the individual coordinates obtained from the three dimensions of the PCA. What’s the point of the PCA then if not to summarize information? As far as I understand correctly, the model should look like: latency = probe location * aggressiveness + probe location * submissiveness + probe location * activity. If yes, then why do we have 3 variables that were used to assess the trait “aggressiveness” tested separately? Also, it seems that the authors are using different terms for the same variables and it makes it difficult to follow. So maybe I am just getting confused here.

Response: We have revised this section to clarify that 10 behaviors were compressed using PCA to create 3 composite traits. This way, only one trait score for “aggressiveness” among the sows was compared to approach latencies (instead of 6 separate behavioral comparisons to approach latencies [i.e., bite, chase, displace, etc.]). The behavior of a sow during the ‘litter restraint test’ was removed from the PCA, as it is evaluated as a separate behavioral measure. We have added to this section, “There were no significant effects of the submissive or active/exploratory trait scores on the latency to approach positive, ambiguous or negative stimulus.” 

Figure 4 could also be improved. R2 are missing and usually regressions are represented using squared frame (same width and height) so that regression lines are easier to interpret.

Response: The graphs of Fig 4 have been revised, and now include r-squared values.

Looking at the relationship between aggressiveness and JB, I wonder if the authors considered a quadratic model? As displayed it feels like more aggressive sows were more optimistic but less aggressive ones were either optimistic or pessimistic. Thus, not a true linear relationship that is consistent with what Asher found if my memories are correct. This point needs to be discussed.

Response: Asher et al reported interactions between cue location, personality trait and housing type, but all were described with a simple linear mixed model. We appreciate the reviewer’s point about the variability/noisiness of the latency response as the aggressive/dominant trait score decreases. We believe that given this variability we would need significantly more observations to successfully fit a more complicated model such a quadratic and thus opted to use a simple linear mixed model as others like Asher et al have done before. 

Discussion

“Both positive and negative judgement bias”. It feels odd to phrase it this way (see previous comment). I would suggest that the authors change for individual differences in judgment bias (here and elsewhere in the paper) for two reasons: 1) we don’t know when a positive and a negative bias starts. 2) it puts a lot of emphasis on the extremes and tend to ignore the four sows that expressed true intermediate responses. How should they be considered? as neutral judgment bias? Using “inter-individual differences” will solve these issues and be more consistent with the literature.

Response: We have also replaced all terms of ‘positive or negative bias’ with the terms “optimistic or pessimistic bias” to be consistent with the literature. We have also included a statement about the ‘undetermined bias’ sows from our study in this section.

“Perhaps surprisingly, we did not find that animals with more severe physical indicators of poor welfare, such as reduced body condition or increased lesion severity, displayed more negative judgement biases”

Well is this really surprising considering that the authors just explained that lameness and BCS were not really variable in their cohort? These two elements should be removed from all conclusion sentences as the authors don’t know whether more lame or more thin animals would express negative judgment bias. The authors could add a sentence stating that “variation in BCS and Lameness was too small for meaningful analyses” and only comment on the severity of the lesions.

Response: We have removed the emphasis on body condition and lameness as indicators of welfare condition, and included the suggested statement of the lack variation of these measures.

L428 is this a continuum though? The authors’ data suggest that submissiveness and aggressiveness are independent (on two axes of the PCA). This is consistent with recent research on dominance in sows, which should be incorporated here.

Response: The word ‘continuum’ has been removed from this sentence, and previous studies which found that lesion scores among sows is not a direct proxy for aggression measurement have been cited.

“Taken together, these results suggest that the judgement bias paradigm used in the present study more likely was evaluating a sow’s response to risk as it relates to consistent individual differences in behavior (i.e., personality) rather than exclusively evaluating the subjective mood state (i.e., mental welfare) of the sow” How do you know? Maybe the “personality differences” you assessed reflected or were affected by the overall mood states of the animals.

I’d like the authors to integrate the growing research that explore judgment biases as states and traits. See research being done in dairy calves for more information on this aspect. I think this will help the authors phrase their results.

Response: We have included more literature which examines judgment bias and personality in other species throughout the discussion. In addition, we have included a short review of the theories behind individual differences in appraisal of stimuli as it relates to state (mood) and trait (affective style) (i.e., Faustino et al., 2015). 

“locomotion-based” well this type of test is typically referred to as a spatial learning task.

Response: These phrase ‘locomotion based’ has been replace with “spatial learning task”.

L436 – 458 I agree with the authors. Similar issues were found in laboratory animals when the relationship between stereotypic behaviours and response to jbt were assessed.

Response: We appreciate the reviewer’s recognition of the relevance for this section.

L460 “(rats, dogs, parrots)” but you cite studies in pigs, dogs and primates.

Response: This sentence has been removed.

L461 Which personality traits though? To my knowledge none of these studies looked at aggressiveness per se. In addition, there is much more in the literature than these three studies and it may help the authors to look more deeply at what has been found in species such as rats, dairy calves, dolphins and many others. Some of these studies specifically looked at dominance, social behaviours, fearfulness etc. All these elements could help the authors better interpreting their results.

Response: This sentence has been removed. 

I also want to attract the attention of the authors that some work being done in dogs and primates are harder to interpret given that questionnaires are sometimes used in dogs and low sample size are frequent in primates. These methodological differences may render comparisons difficult.

Response: We agree with the reviewer, that personality determined from human ratings, rather than ethological coding, can be difficult to interpret (or generalize to other animal personality studies). Therefore, we have reduced the reference to such studies and focused on referring to studies with similar methodology as our present study.

L464 to 473. It is a little difficult to follow the authors here. Please try to rewrite and clarify.

Response: This paragraph has been rewritten to clarify our point that there are themes within the behavioral syndrome, coping style, and personality literature which link the traits related to survival (i.e. large risk taking for big reward). 

L476 why not, but do the authors have evidence that injuries lead to small differences in subjective state?

Response: We have removed this section of the sentence to prevent the insinuation that we assume small injuries would lead to differences in mood state.

L480 yes! Thus, I am sorry to say that calling the behavioural assessment done in these sows “personality traits” is a bit misleading (see previous comments), unless the authors can better justify?

Response: We have replaced the word ‘personality traits’ with ‘behavioral traits’ throughout the manuscript, but maintain the theme of individual differences in behavior consistent over time were found with the dataset; as we have established this difference using the same study cohort in another peer reviewed study (Horback and Parsons, 2018, Behavioral Processes).

L511 a very recent study in mice support the point being made here (see Jardim et al., 2021)

Response: The Jardim et al (2021) paper has been incorporated into this sentence.

L516 yes! You will see that following studies in calves from the same group explored the logic you described at the end of the discussion.

Response: We have incorporated additional studies on animal personality, judgment bias, and state vs trait of appraisal analysis throughout the introduction and discussion based on the recommendations of the reviewer.

Attachment Submitted filename: PLOS ONE revision note CLEAN.docx Click here for additional data file.

# Peer review: Decision Letter 1

**DOI:** 10.1371/journal.pone.0264258.r003

**Authors:** Miquel Llorente

22 Nov 2021

PONE-D-21-16018R1Judgement bias of group housed gestating sows predicted by behavioral traits, but not physical measures of welfarePLOS ONE

Dear Dr. Horback,

Thank you for submitting your manuscript to PLOS ONE. After careful consideration, we feel that it has merit but does not fully meet PLOS ONE’s publication criteria as it currently stands. Therefore, we invite you to submit a revised version of the manuscript that addresses the points raised during the review process.

Please submit your revised manuscript by Jan 06 2022 11:59PM. If you will need more time than this to complete your revisions, please reply to this message or contact the journal office at plosone@plos.org. When you're ready to submit your revision, log on to https://www.editorialmanager.com/pone/ and select the 'Submissions Needing Revision' folder to locate your manuscript file.

Please include the following items when submitting your revised manuscript:

- A rebuttal letter that responds to each point raised by the academic editor and reviewer(s). You should upload this letter as a separate file labeled 'Response to Reviewers'.
- A marked-up copy of your manuscript that highlights changes made to the original version. You should upload this as a separate file labeled 'Revised Manuscript with Track Changes'.
- An unmarked version of your revised paper without tracked changes. You should upload this as a separate file labeled 'Manuscript'.

If you would like to make changes to your financial disclosure, please include your updated statement in your cover letter. Guidelines for resubmitting your figure files are available below the reviewer comments at the end of this letter.

If applicable, we recommend that you deposit your laboratory protocols in protocols.io to enhance the reproducibility of your results. Protocols.io assigns your protocol its own identifier (DOI) so that it can be cited independently in the future. For instructions see: https://journals.plos.org/plosone/s/submission-guidelines#loc-laboratory-protocols. Additionally, PLOS ONE offers an option for publishing peer-reviewed Lab Protocol articles, which describe protocols hosted on protocols.io. Read more information on sharing protocols at https://plos.org/protocols?utm_medium=editorial-email&utm_source=authorletters&utm_campaign=protocols.

We look forward to receiving your revised manuscript.

Kind regards,

Miquel Llorente, PhD

Academic Editor

PLOS ONE

Journal Requirements:

Please review your reference list to ensure that it is complete and correct. If you have cited papers that have been retracted, please include the rationale for doing so in the manuscript text, or remove these references and replace them with relevant current references. Any changes to the reference list should be mentioned in the rebuttal letter that accompanies your revised manuscript. If you need to cite a retracted article, indicate the article’s retracted status in the References list and also include a citation and full reference for the retraction notice.

[Note: HTML markup is below. Please do not edit.]

Reviewers' comments:

Reviewer's Responses to Questions

**Comments to the Author**

1. If the authors have adequately addressed your comments raised in a previous round of review and you feel that this manuscript is now acceptable for publication, you may indicate that here to bypass the “Comments to the Author” section, enter your conflict of interest statement in the “Confidential to Editor” section, and submit your "Accept" recommendation.

Reviewer #2: (No Response)

**********

2. Is the manuscript technically sound, and do the data support the conclusions?

The manuscript must describe a technically sound piece of scientific research with data that supports the conclusions. Experiments must have been conducted rigorously, with appropriate controls, replication, and sample sizes. The conclusions must be drawn appropriately based on the data presented. 

Reviewer #2: Yes

**********

3. Has the statistical analysis been performed appropriately and rigorously? 

Reviewer #2: Yes

**********

4. Have the authors made all data underlying the findings in their manuscript fully available?

The PLOS Data policy requires authors to make all data underlying the findings described in their manuscript fully available without restriction, with rare exception (please refer to the Data Availability Statement in the manuscript PDF file). The data should be provided as part of the manuscript or its supporting information, or deposited to a public repository. For example, in addition to summary statistics, the data points behind means, medians and variance measures should be available. If there are restrictions on publicly sharing data—e.g. participant privacy or use of data from a third party—those must be specified.

Reviewer #2: Yes

**********

5. Is the manuscript presented in an intelligible fashion and written in standard English?

PLOS ONE does not copyedit accepted manuscripts, so the language in submitted articles must be clear, correct, and unambiguous. Any typographical or grammatical errors should be corrected at revision, so please note any specific errors here.

Reviewer #2: Yes

**********

6. Review Comments to the Author

Please use the space provided to explain your answers to the questions above. You may also include additional comments for the author, including concerns about dual publication, research ethics, or publication ethics. (Please upload your review as an attachment if it exceeds 20,000 characters)

Reviewer #2: First, I want to thank the authors for a great revision of their paper. I think it has greatly improved. I also think the study contributes to highlight a growing problem in the field: what do we do with animal welfare markers that do not correlate? I would like the authors to consider putting more emphasis on this point rather than concluding that JBTs provide limited information. We actually do not know which marker is the most limited. It is also interesting to put forward that when JBT are used in absence of an emotional manipulation, it typically provides information about individual characteristics. I made a few more comments that I’d like the authors to consider (see below).

Abstract:

The authors make a very strong claim here (i.e., that JBT may not “be the most appropriate method for evaluating affective state”), but I am not convinced. This argument is based on 1) high variability in responses to JBT in enriched animals, 2) a positive relationship with aggressiveness and 3) a lack of relationship with other so-called welfare markers (e.g., mostly the number of lesions after regrouping).

First, yes, there is high variability, but this does not mean swine kept in barren conditions would not score worse.

Second, yes it correlates with aggressiveness, which makes sense given that aggressiveness often correlates with proactive coping styles, impulsivity and optimism. I’m not sure if the authors saw this recent review (Lecorps et al., 2021. Negative expectations and vulnerability to stressors in animals) but it seems very relevant for this study!

Third, are these physical indicators truly reflecting what animals feel or their welfare state? Are only unhealthy humans feeling terrible?

I think the authors should discuss more this aspect because it is a very important one and they are not the only ones that failed to show correlations between psychological and physical markers of well-being (e.g., Lecorps et al. 2021. Regrouping induces anhedonia-like responses in dairy heifers; in that study no correlations between anhedonia-like behaviours and the number of agonistic interactions received during regrouping were found).

Importantly, I think this does not necessarily mean that the methodologies do not work well but rather that our conception of welfare is somewhat challenged. I would like the authors to include this aspect (i.e., the difficulty to correlate different welfare parameters) in their conclusions. As is, the claims are not largely supported by the authors’ dataset.

L54 There are several reviews addressing cumulative affective experiences and mood-related disorders in farm animals (Poirier et al., 2019, Bateson and Poirier, 2019, Lecorps et al., 2021, McLellan and Mason, 2021), maybe worth mentioning at least one of these so that the interested reader can learn more?

L69 to 73 I haven’t read this particular contribution of Stamps but I find some of the arguments quite surprising. For instance, what do “early in life” or “biologically based” mean? Are all traits more clearly expressed in “novel and unpredictable situations” or just the ones we care to measure in animals? It makes perfect sense if we assess neophobia, boldness or pessimism but does it make sense for sociability and aggressiveness?

In my opinion, these are risky claims, but the authors are of course free to keep this section if they feel it helps with the narrative.

L97 “Given that there is limited information on the use and validity of

judgement bias testing to evaluate affective states in sexually-mature sows, there is a need to investigate possible influences on performance in this cognitive task. As greater emphasis is being placed on the welfare of commercial sows, because they live the longest out of all production swine and thus have the greatest capacity to incur chronic suffering if their welfare is compromised, investigating new tools for welfare assessment is crucial.”

The second sentence could be deleted as it basically repeats what is said earlier (L85).

I’m still not sure to understand the aims as currently formulated.

L102 to 108 maybe this part belongs to the method section?

L112 “if judgement bias reflects the affective state of an animal (i.e., biological health and mental state), then it follows that physical measurements of welfare (i.e., skin lesions, body condition) should also impact performance in judgement bias task”

As argued before this may not be necessarily true. However this is a good working hypothesis.

L278 For clarity sake I would start this sentence by “Additional probe cues”.

L358 to 363 this basically repeats the previous sentences, delete?

Figure 3 seems to have changed based on caption changes but I could not access it (neither any other figures).

Figure 4 has also been changed according to the authors’ response, but I could not see the changes.

L446 affective or coping style?

L454 “we need to reconcile inconsistencies when comparing the physical and assumed psychological welfare state.” I’d push the authors to consider that these may not be inconsistencies. Health being a poor predictor of psychological well-being in humans, it may well be the same in animals. However, I agree with the authors that lameness should, in theory, correlate with judgment bias given its severe impact on the animals’ quality of life.

L479 there is a problem with this sentence (missing word?).

L519 “it can be argued”. There are several typos along the manuscript that the authors will want to address before publication.

**********

7. PLOS authors have the option to publish the peer review history of their article (what does this mean?). If published, this will include your full peer review and any attached files.

If you choose “no”, your identity will remain anonymous but your review may still be made public.

**Do you want your identity to be public for this peer review?** For information about this choice, including consent withdrawal, please see our Privacy Policy.

Reviewer #2: No

[NOTE: If reviewer comments were submitted as an attachment file, they will be attached to this email and accessible via the submission site. Please log into your account, locate the manuscript record, and check for the action link "View Attachments". If this link does not appear, there are no attachment files.]

While revising your submission, please upload your figure files to the Preflight Analysis and Conversion Engine (PACE) digital diagnostic tool, https://pacev2.apexcovantage.com/. PACE helps ensure that figures meet PLOS requirements. To use PACE, you must first register as a user. Registration is free. Then, login and navigate to the UPLOAD tab, where you will find detailed instructions on how to use the tool. If you encounter any issues or have any questions when using PACE, please email PLOS at figures@plos.org. Please note that Supporting Information files do not need this step.

# Author comment: Author response to Decision Letter 1

**DOI:** 10.1371/journal.pone.0264258.r004

31 Dec 2021

Revision note

12/28/2021

PONE-D-21-16018

Judgement bias of group housed gestating sows predicted by personality traits, but not physical measures of welfare

Reviewer #2: First, I want to thank the authors for a great revision of their paper. I think it has greatly improved. I also think the study contributes to highlight a growing problem in the field: what do we do with animal welfare markers that do not correlate? I would like the authors to consider putting more emphasis on this point rather than concluding that JBTs provide limited information. We actually do not know which marker is the most limited. It is also interesting to put forward that when JBT are used in absence of an emotional manipulation, it typically provides information about individual characteristics. I made a few more comments that I’d like the authors to consider (see below).

Response: We thank the reviewer for providing very helpful feedback to improve this manuscript. We agree that different welfare metrics will have different limitations and is a challenge in elucidating the overall affective state of the animal. In the revised version of this manuscript, we suggest that individual behavioral characteristics could create differential emotional responses that are captured in the JBT. Perhaps an animal’s boldness or risk attributes determine how they perceive a complicated social environment like pen gestation and as such influences their emotional response. We have amended the introduction and discussion to place more emphasis on the role of cognitive bias testing; that is, how do we interpret response latency given the possibility that different factors (eg behavioral traits and lesion) may be differentially contributing to the animal’s affective state. 

Abstract:

The authors make a very strong claim here (i.e., that JBT may not “be the most appropriate method for evaluating affective state”), but I am not convinced. This argument is based on 1) high variability in responses to JBT in enriched animals, 2) a positive relationship with aggressiveness and 3) a lack of relationship with other so-called welfare markers (e.g., mostly the number of lesions after regrouping).

First, yes, there is high variability, but this does not mean swine kept in barren conditions would not score worse. Second, yes it correlates with aggressiveness, which makes sense given that aggressiveness often correlates with proactive coping styles, impulsivity and optimism. I’m not sure if the authors saw this recent review (Lecorps et al., 2021. Negative expectations and vulnerability to stressors in animals) but it seems very relevant for this study! Third, are these physical indicators truly reflecting what animals feel or their welfare state? Are only unhealthy humans feeling terrible?

Response: Not sure, what the reviewer means in terms of sows in barren conditions scoring worse. Our goal is not the response of the group but that fact that several individual animals essentially interpret the ambiguous stimulus as the negative cue. I do not know how these animals could score worse even if housed in barren conditions. However, this does speaks towards the notion that personality attributes could be creating the emotional experience captured in this JBT. We also agree that physical compromise could, but necessarily, result in negative affective states and tried to better explain the observed disconnect between lesions scores and JBT outcomes. The abstract has been modified to capture these nuances.

I think the authors should discuss more this aspect because it is a very important one and they are not the only ones that failed to show correlations between psychological and physical markers of well-being (e.g., Lecorps et al. 2021. Regrouping induces anhedonia-like responses in dairy heifers; in that study no correlations between anhedonia-like behaviours and the number of agonistic interactions received during regrouping were found). Importantly, I think this does not necessarily mean that the methodologies do not work well but rather that our conception of welfare is somewhat challenged. I would like the authors to include this aspect (i.e., the difficulty to correlate different welfare parameters) in their conclusions. As is, the claims are not largely supported by the authors’ dataset.

Response: We appreciate the reviewer’s request to make this point more explicit in our paper. We have added the following lines to our discussion to highlight that this disconnect between physical and psychological welfare assessments (lines 441-449): “Current welfare assessments for breeding sows focus on physical ailments (e.g., lameness, body condition and skin lesions), as well as being the target of aggressive behaviors (e.g., bites, scratches, displacement). However, given that recent work in dairy heifer welfare has reported a lack of correlation between behavioral indicators of anhedonia and the number of agonistic interactions received during regrouping (Lecorps et al., 2021), there is a need to carefully critique our assumptions of how welfare is experienced by the animal. If we are using judgement bias testing as an indicator of subjective mental welfare of animals, we also need to better understand how, and if, the degree and duration of physical comprise contribute to an animal’s psychological welfare state.”

L54 There are several reviews addressing cumulative affective experiences and mood-related disorders in farm animals (Poirier et al., 2019, Bateson and Poirier, 2019, Lecorps et al., 2021, McLellan and Mason, 2021), maybe worth mentioning at least one of these so that the interested reader can learn more?

Response: We have revised this sentence to clarify that little attention is currently being given to breeding sow affective state (and not farm animals in general), but we have added a citation to this sentence to allow the reader to know of the growth of knowledge on the subject of affective states in farm animals. 

L69 to 73 I haven’t read this particular contribution of Stamps but I find some of the arguments quite surprising. For instance, what do “early in life” or “biologically based” mean? Are all traits more clearly expressed in “novel and unpredictable situations” or just the ones we care to measure in animals? It makes perfect sense if we assess neophobia, boldness or pessimism but does it make sense for sociability and aggressiveness? In my opinion, these are risky claims, but the authors are of course free to keep this section if they feel it helps with the narrative.

Response: This summary of personality has been revised to reduce any claims of genetically-linked traits, or strict consistency over an organism’s lifetime. This sentence now reads, “Personality can be described as characteristics of behavioral reactions such as intensity, speed, response threshold, latency and recovery time, which are most clearly expressed in novel and unpredictable situations, and are relatively stable throughout an individual’s development.”

L97 “Given that there is limited information on the use and validity of judgement bias testing to evaluate affective states in sexually-mature sows, there is a need to investigate possible influences on performance in this cognitive task. As greater emphasis is being placed on the welfare of commercial sows, because they live the longest out of all production swine and thus have the greatest capacity to incur chronic suffering if their welfare is compromised, investigating new tools for welfare assessment is crucial.”

The second sentence could be deleted as it basically repeats what is said earlier (L85). I’m still not sure to understand the aims as currently formulated.

Response: The second sentence has been removed to prevent redundancy. A sentence clarifying the aim of this study has been added in its place.

L102 to 108 maybe this part belongs to the method section?

Response: These sentences have been removed as they summarize the methods of this study.

L112 “if judgement bias reflects the affective state of an animal (i.e., biological health and mental state), then it follows that physical measurements of welfare (i.e., skin lesions, body condition) should also impact performance in judgement bias task”

As argued before this may not be necessarily true. However this is a good working hypothesis.

Response: We have kept this portion of the hypothesis in place, as we agree with the reviewer that physical health may not be the best predictor of subjective mental state; this notion is assumed in some animal emotion/welfare literature.

L278 For clarity sake I would start this sentence by “Additional probe cues”.

Response: This change has been made.

L358 to 363 this basically repeats the previous sentences, delete?

Response: These sentences have been deleted.

Figure 3 seems to have changed based on caption changes but I could not access it (neither any other figures).

Response: We do not know why the reviewer could not access the new Figure 3.

Figure 4 has also been changed according to the authors’ response, but I could not see the changes.

Response: We do not know why the reviewer could not access the new Figure 4.

L446 affective or coping style?

Response: This change has been made.

L454 “we need to reconcile inconsistencies when comparing the physical and assumed psychological welfare state.” I’d push the authors to consider that these may not be inconsistencies. Health being a poor predictor of psychological well-being in humans, it may well be the same in animals. However, I agree with the authors that lameness should, in theory, correlate with judgment bias given its severe impact on the animals’ quality of life.

Response: We agree with the reviewer on this suggestion, and have added a sentence the following sentence, “If we are using judgement bias testing as an indicator of subjective mental welfare of animals, we also need to better understand how, and if, the degree and duration of physical comprise contribute to an animal’s psychological welfare state.”

L479 there is a problem with this sentence (missing word?).

Response: This sentence has been removed.

L519 “it can be argued”. There are several typos along the manuscript that the authors will want to address before publication.

Response: The word “be” has been added.

Attachment Submitted filename: PLOS ONE revision note #3 CLEAN.docx Click here for additional data file.

# Peer review: Decision Letter 2

**DOI:** 10.1371/journal.pone.0264258.r005

**Authors:** Miquel Llorente

8 Feb 2022

Judgement bias of group housed gestating sows predicted by behavioral traits, but not physical measures of welfare

PONE-D-21-16018R2

Dear Dr. Horback,

We’re pleased to inform you that your manuscript has been judged scientifically suitable for publication and will be formally accepted for publication once it meets all outstanding technical requirements.

Within one week, you’ll receive an e-mail detailing the required amendments. When these have been addressed, you’ll receive a formal acceptance letter and your manuscript will be scheduled for publication.

An invoice for payment will follow shortly after the formal acceptance. To ensure an efficient process, please log into Editorial Manager at http://www.editorialmanager.com/pone/, click the 'Update My Information' link at the top of the page, and double check that your user information is up-to-date. If you have any billing related questions, please contact our Author Billing department directly at authorbilling@plos.org.

If your institution or institutions have a press office, please notify them about your upcoming paper to help maximize its impact. If they’ll be preparing press materials, please inform our press team as soon as possible -- no later than 48 hours after receiving the formal acceptance. Your manuscript will remain under strict press embargo until 2 pm Eastern Time on the date of publication. For more information, please contact onepress@plos.org.

Kind regards,

Miquel Llorente, PhD

Academic Editor

PLOS ONE

Reviewers' comments:

Reviewer's Responses to Questions

**Comments to the Author**

1. If the authors have adequately addressed your comments raised in a previous round of review and you feel that this manuscript is now acceptable for publication, you may indicate that here to bypass the “Comments to the Author” section, enter your conflict of interest statement in the “Confidential to Editor” section, and submit your "Accept" recommendation.

Reviewer #2: All comments have been addressed

**********

2. Is the manuscript technically sound, and do the data support the conclusions?

The manuscript must describe a technically sound piece of scientific research with data that supports the conclusions. Experiments must have been conducted rigorously, with appropriate controls, replication, and sample sizes. The conclusions must be drawn appropriately based on the data presented. 

Reviewer #2: Yes

**********

3. Has the statistical analysis been performed appropriately and rigorously? 

Reviewer #2: Yes

**********

4. Have the authors made all data underlying the findings in their manuscript fully available?

The PLOS Data policy requires authors to make all data underlying the findings described in their manuscript fully available without restriction, with rare exception (please refer to the Data Availability Statement in the manuscript PDF file). The data should be provided as part of the manuscript or its supporting information, or deposited to a public repository. For example, in addition to summary statistics, the data points behind means, medians and variance measures should be available. If there are restrictions on publicly sharing data—e.g. participant privacy or use of data from a third party—those must be specified.

Reviewer #2: Yes

**********

5. Is the manuscript presented in an intelligible fashion and written in standard English?

PLOS ONE does not copyedit accepted manuscripts, so the language in submitted articles must be clear, correct, and unambiguous. Any typographical or grammatical errors should be corrected at revision, so please note any specific errors here.

Reviewer #2: Yes

**********

6. Review Comments to the Author

Please use the space provided to explain your answers to the questions above. You may also include additional comments for the author, including concerns about dual publication, research ethics, or publication ethics. (Please upload your review as an attachment if it exceeds 20,000 characters)

Reviewer #2: (No Response)

**********

7. PLOS authors have the option to publish the peer review history of their article (what does this mean?). If published, this will include your full peer review and any attached files.

If you choose “no”, your identity will remain anonymous but your review may still be made public.

**Do you want your identity to be public for this peer review?** For information about this choice, including consent withdrawal, please see our Privacy Policy.

Reviewer #2: No

# Editor report: Acceptance letter

**DOI:** 10.1371/journal.pone.0264258.r006

**Authors:** Miquel Llorente

11 Feb 2022

PONE-D-21-16018R2 

Judgement bias of group housed gestating sows predicted by behavioral traits, but not physical measures of welfare 

Dear Dr. Horback:

I'm pleased to inform you that your manuscript has been deemed suitable for publication in PLOS ONE. Congratulations! Your manuscript is now with our production department. 

If your institution or institutions have a press office, please let them know about your upcoming paper now to help maximize its impact. If they'll be preparing press materials, please inform our press team within the next 48 hours. Your manuscript will remain under strict press embargo until 2 pm Eastern Time on the date of publication. For more information please contact onepress@plos.org.

If we can help with anything else, please email us at plosone@plos.org. 

Thank you for submitting your work to PLOS ONE and supporting open access. 

Kind regards, 

PLOS ONE Editorial Office Staff

on behalf of

Dr. Miquel Llorente 

Academic Editor

PLOS ONE
